# Nimbolide protects against endotoxin-induced acute respiratory distress syndrome by inhibiting TNF-α mediated NF-κB and HDAC-3 nuclear translocation

**DOI:** 10.1038/s41419-018-1247-9

**Published:** 2019-01-28

**Authors:** Venkatesh Pooladanda, Sowjanya Thatikonda, Swarna Bale, Bijay Pattnaik, Dilep Kumar Sigalapalli, Nagendra Babu Bathini, Shashi Bala Singh, Chandraiah Godugu

**Affiliations:** 1 Department of Regulatory Toxicology, National Institute of Pharmaceutical Education and Research (NIPER), Balanagar, Hyderabad, Telangana 500037 India; 2 Centre of Excellence in Asthma & Lung Disease and Molecular Immunogenetics Laboratory, CSIR-Institute of Genomics and Integrative Biology, 110007 New Delhi, India; 3 Department of Medicinal Chemistry, National Institute of Pharmaceutical Education and Research (NIPER), Balanagar, Hyderabad, Telangana 500037 India

## Abstract

Acute respiratory distress syndrome (ARDS) is characterized by an excessive acute inflammatory response in lung parenchyma, which ultimately leads to refractory hypoxemia. One of the earliest abnormalities seen in lung injury is the elevated levels of inflammatory cytokines, among them, the soluble tumor necrosis factor (TNF-α) has a key role, which exerts cytotoxicity in epithelial and endothelial cells thus exacerbates edema. The bacterial lipopolysaccharide (LPS) was used both in vitro (RAW 264.7, THP-1, MLE-12, A549, and BEAS-2B) and in vivo (C57BL/6 mice), as it activates a plethora of overlapping inflammatory signaling pathways involved in ARDS. Nimbolide is a chemical constituent of *Azadirachta indica*, which contains multiple biological properties, while its role in ARDS is elusive. Herein, we have investigated the protective effects of nimbolide in abrogating the complications associated with ARDS. We showed that nimbolide markedly suppressed the nitrosative-oxidative stress, inflammatory cytokines, and chemokines expression by suppressing iNOS, myeloperoxidase, and nitrotyrosine expression. Moreover, nimbolide mitigated the migration of neutrophils and mast cells whilst normalizing the LPS-induced hypothermia. Also, nimbolide modulated the expression of epigenetic regulators with multiple HDAC inhibitory activity by suppressing the nuclear translocation of NF-κB and HDAC-3. We extended our studies using molecular docking studies, which demonstrated a strong interaction between nimbolide and TNF-α. Additionally, we showed that treatment with nimbolide increased GSH, Nrf-2, SOD-1, and HO-1 protein expression; concomitantly abrogated the LPS-triggered TNF-α, p38 MAPK, mTOR, and GSK-3β protein expression. Collectively, these results indicate that TNF-α-regulated NF-κB and HDAC-3 crosstalk was ameliorated by nimbolide with promising anti-nitrosative, antioxidant, and anti-inflammatory properties in LPS-induced ARDS.

## Introduction

Acute respiratory distress syndrome (ARDS) is a life-threatening disease caused by shock, sepsis, and pneumonia, which eventually culminates into multiple organ failure^1^. ARDS is one of the major cause of morbidity and mortality across the world and the epidemiological data suggest that there are 18–79 ARDS cases among 1,00,000 persons per year^2^. Indeed, there is a certain scope to treat infectious lung diseases for reducing the mortality rate^3^. Lipopolysaccharide (LPS) binds to its cognate Toll-like receptor-4 (TLR-4) and the co-receptor cluster of differentiation 14 (CD14) results in lung parenchymal damage, neutrophil accumulation in the interstitial and alveolar compartments, elevated vascular permeability, provocation of pulmonary edema and fibrin deposition^4,5^. LPS stimulation initiates multiple molecular intracellular signaling events, including classical nuclear factor-κB (NF-κB) activation, thereby promoting translocation into the nucleus, thus release inflammatory cytokines principally interleukins (IL-1β, IL-2, and IL-6) and chemokines (macrophage inflammatory proteins, MIP-1α/β)^6^. On the other side, TLR-4 activation enhances the tumor necrosis factor-α (TNF-α) production, which is a pleiotropic cytokine of the TNF superfamily involves in the pathogenesis of various inflammatory diseases by inducing the oxidative stress, while depleting antioxidant levels^7^. Therefore, either suppressing the TNF-α secretion or obstruction its biological actions by pharmacological modulators might have eminent therapeutic potential in treating various inflammatory lung diseases^8^.

Histone deacetylases (HDACs) play a crucial role in various diseases, including cancer, diabetes, cardiovascular, neurological, and inflammatory diseases. HDACs are of different classes, among them, HDAC-1 has an important role in inflammatory diseases, where it was found to negatively regulate the inflammatory signaling to repress the expression of NF-κB regulated genes. Whereas, HDAC-2 does not involve in the NF-κB signaling directly, but it regulates the NF-κB activity in association with HDAC-1^9^. On the other side, it was reported that HDAC-1 inhibition in intestinal epithelial cells leads to an increase in p65 NF-κB phosphorylation and nuclear localization^10^. The role of HDAC-3 was extensively studied by Elisabeth Ziesché et al. and revealed its importance as a co-activator in IL-1-induced inflammatory signaling mediated by the removal of inhibitory acetyl groups from p65 NF-κB^11^. Similarly, Niek GJ Leus et al. used RGFP966 (HDAC-3 selective inhibitor) in pulmonary inflammation model and found reduced NF-κB transcription with a reduction in the expression of IL-1β, IL-6, and IL-12b cytokines in macrophages and found an increase in the expression of the anti-inflammatory cytokine IL-10^12^; while HDAC-4 involves in ROS generation via enhanced vascular cell adhesion protein 1 (VCAM-1) expression^13^. Collectively, it infers that HDAC inhibitors maintain the balance between pro- and anti-inflammatory gene expression, thereby suppress the lung inflammation^14,15^. Thus, HDAC inhibitors have profound scope in treating various inflammatory diseases.

Nimbolide, a natural chemical constituent isolated from the leaves and flowers of neem (*Azadirachta indica*). It manifested assorted biological activities, including antimalarial, antibacterial, anticancer, and anti-inflammatory activities^16^. In the current study, to our knowledge, we show for the first time the therapeutic potential and molecular mechanism of nimbolide in LPS-induced ARDS-associated pleural inflammation in mice and the underlying molecular inflammatory events were recapitulated in cultured macrophages and lung epithelial cells. Notably, our data demonstrate that nimbolide selectively suppressed IκB-α-regulated p65 NF-κB and HDAC-3 crosstalk by inhibiting TNF-α in LPS-induced ARDS.

## Materials and methods

### Materials

Nimbolide was purchased from Aptus therapeutics, Hyderabad, India. TNF-α recombinant protein was purchased from Thermo Fisher Scientific, USA. LPS from *Escherichia coli* (055: B5), and phorbol 12-myristate 13-acetate (PMA), 3-(4,5-Dimethylthiazol-2-yl)-2,5-Diphenyltetrazolium Bromide (MTT), dimethyl sulfoxide (DMSO), Griess reagent, 2',7'–dichlorofluorescin diacetate (DCFDA), 4ʹ, 6ʹ-diamidino-2-phenylindole (DAPI), ethylenediaminetetraacetic acid (EDTA), hematoxylin, eosin, toulidine blue (TB), Ehrlich reagent, Giemsa stain, reduced glutathione (GSH), bovine serum albumin (BSA), sodium dodecyl sulphate (SDS), glacial acetic acid, sodium nitrite and bicinchoninic acid (BCA) reagent were purchased from Sigma-Aldrich, USA. Anti-nitrotyrosine, anti-Nrf-2, anti-SOD-1, anti-HO-1, anti-TNF-α, anti-PCNA, and anti-β-Actin antibodies were purchased from Santa Cruz Biotechnology, USA. Anti-iNOS antibody was purchased from Sigma-Aldrich, USA. Anti-p-NF-κB (p-p65), anti-NF-κB (p65), anti-p-IKKα/β, anti-p-IκB, anti-IκB, anti-p-MAPK p38, anti-MAPK p38, anti-p-GSK-3β, anti-GSK-3β, anti-mTOR, anti-HDAC-1, anti-HDAC-2, anti-HDAC-3, anti-HDAC-4, and anti-H3 antibodies were purchased from Cell Signaling Technologies, USA. Anti-MPO antibody was purchased from PathnSitu Biotechnologies, USA. All secondary anti-rabbit, anti-goat, and anti-mouse antibodies were purchased from Santa Cruz Biotechnology, USA. TNF-α siRNA was purchased from Dharmacon™, USA. All other chemicals were of analytical grade and obtained commercially.

### Cell culture

RAW 264.7 (murine macrophages) and A549 (human type II alveolar epithelial cells) cells were obtained from National Centre for Cell Science (NCCS), Pune, India. These cell lines were cultured in appropriate Dulbecco's Modified Eagle's medium (DMEM) and Roswell Park Memorial Institute medium (RPMI-1640) (Invitrogen, USA), respectively. MLE-12 (mouse lung epithelial cells) and BEAS-2B (human bronchial lung epithelial cells) cell lines were procured from American Type Culture Collection (ATCC), USA and cultured in 1:1 ratio of low glucose DMEM and Ham's F-12K (Kaighn's) medium (Invitrogen, USA). The human monocytic cell line, THP-1 was a kind gift from Dr. Sanjeev Khosla (Lab of Mammalian Genetics, Centre for DNA Fingerprinting and Diagnostics (CDFD), Hyderabad, India) and these cells were grown in RPMI-1640 medium. All the cells were supplemented with 10% fetal bovine serum and 1% antibiotic-antimycotic solution (Sigma-Aldrich, USA). Cells were grown in a humidified CO_2_ incubator at 37 °C temperature. For differentiating the monocytes into macrophages, THP-1 cells were primed with PMA (5 nM) for 48 h.

### Measurement of cell viability

Cell viability was determined by MTT assay as described previously with slight modifications^17^. Here, the cells were seeded in 96-well plate and treated with nimbolide (0.5-10 µM) for 24 h. Then cells were washed with PBS and MTT (0.5 mg/ml) was added to each well, followed by formazan crystals were solubilized with DMSO and absorbance was measured at 570 nm with the spectrophotometer (Spectra Max, M4 Molecular devices, USA).

### Measurement of cellular ROS levels

The ROS levels were measured by DCFDA (Sigma-Aldrich, USA) and MitoSOX Red (Invitrogen, USA) fluorescent dyes. For the flow cytometric analysis, RAW 264.7 and differentiated THP-1 cells (1 × 10^5^ cells/well) were seeded in 6-well plate. At 80 % confluence, cells were pre-treated with nimbolide (0.5 and 1 µM) for 24 h. Then cells were stimulated with LPS (1 µg/ml) for 30 min to induce oxidative stress. Later, these cells were further incubated with 10 μM of DCFDA and 5 μM of MitoSOX Red reagent for 15 and 30 min, respectively. After trypsinization, cells were subjected to flow cytometric analysis (BD Accuri C6 flow cytometer, USA) and relative geo-mean was measured. For visualization of apparent changes, cells were observed under Nikon Eclipse inverted fluorescent microscope, (Japan) at ×200 magnification immediately following dye exposure. The fluorescence intensity was measured using a multimode plate reader.

### Immunofluorescence (IF)

After LPS or TNF-α stimulation, cells were washed with PBS and fixed with 4% paraformaldehyde for 5 min at room temperature. Then cells were washed and permeabilized with 0.1% Triton-X. Further, the cells were incubated with blocking buffer (3% BSA) for 1 h and probed with primary antibody overnight at 4 °C. Cells were washed and incubated with secondary antibody for 1 h at room temperature. The primary antibodies and secondary antibodies conjugated to FITC or rhodamine (Sigma-Aldrich, USA) used at 1:200 dilutions. The nuclei were visualized with DAPI staining. The coverslips were mounted on to the chamber glass slides with Fluoroshield™ histology mounting medium (Sigma-Aldrich, USA). Images of the stained slides were captured by Leica TCS SP8 Laser Scanning Spectral Confocal Microscope (Germany).

### HDAC fluorometric assay

HDAC levels upon nimbolide treatment (0.05, 0.1, 1, and 2.5 µM) were determined by Histone Deacetylase Assay Kit, Fluorometric (Sigma-Aldrich, USA) according to manufacturer instructions. The HDAC inhibitor, Trichostatin A was used to compare the nimbolide HDAC inhibitory activity from the standard curve.

### Animals

Male C57BL/6 mice (8 weeks old) were utilized for the experiment and maintained with 12 h dark/light cycle in an animal house at ambient conditions. Mice were acclimatized for 1 week before the study and given free access to food and water *ad libitum*. All the animal studies were conducted under the due endorsement of Institutional Animal Ethics Committee (IAEC) of National Institute of Pharmaceutical Educational and Research (NIPER), Hyderabad, India, as per the Committee for the Purpose of Control and Supervision of Experiments on Animals (CPCSEA) guidelines of Government of India.

### ARDS animal model

Mice were randomly divided into seven groups (*n* = 8): Control, LPS alone (50 µg per mice), LPS + Nimbolide pre-treatment (0.3, 1 and 3 mg/kg), nimbolide alone (3 mg/kg), and concurrent nimbolide treatment group (3 mg/kg nimbolide was given through intraperitoneal (i.p.) administration immediately followed by LPS oropharyngeal instillation for 12 h). The pre-treatment groups received an i.p. injection of nimbolide (0.3, 1 and 3 mg/kg) for 5 days followed by LPS instillation for 12 h except for nimbolide alone group. An equal volume of vehicle, instead of nimbolide was given to the LPS control group. The animals were anesthetized using ketamine (8 mg/kg), and xylazine (45 mg/kg) i.p. administration. When the anesthesia is attained, the animals were placed on a mouse intubation platform at 60^ο^ inclined angle with rubber band running under the upper incisors. The tongue of the mouse was pulled and held with blunt forceps. Then, 50 µg of LPS in 50 µl volume of sterile water for injection was administered into the back of oral cavity using a micropipette, the tongue was held until the liquid disappeared from the mouth while the gasping sound is audible as an indication of instillation into lungs.

### Changes in body weights, lung weight index, and body temperature

Initially. body weights of animals were taken before administration of nimbolide. After 5 days of nimbolide pre-treatment, LPS was instilled to the animals for 12 h and again body weights were measured and net body weight changes were calculated. After animal sacrifice, lung weights were noted to determine the lung weight index. Body temperatures were recorded before and after the administration of LPS with a rectal probe attached to the homeothermic monitor (Harvard Apparatus, USA).

### Bronchoalveolar lavage (BAL) analysis

Mice were sacrificed and the lungs were lavaged 3 times with 1 ml ice-cold PBS. BAL samples were pooled and centrifuged at 300 × *g* for 10 min. The cell pellets obtained after centrifugation were resuspended in 1 ml PBS and subjected to differential cell counter ADVIA 2120i Hematology System (Siemens, Germany).

### Blood analysis

Blood was collected from mice by cardiac puncture to determine different blood parameters. The blood was collected in heparin solution contained centrifugal tubes. The whole blood was subjected to automatic blood cell analyzer for detailed hematological analysis.

### Measurement of nitrite and GSH levels

Nitrite levels were quantified by Griess assay^18^, while glutathione levels were measured by Ellman’s reagent^19^ as described earlier with slight changes.

### Multiplex bead-based cytokine assay

After 12 h of LPS treatment, cytokine and chemokine levels were measured in RAW 264.7 cells and lung tissues by Luminex assay based on xMAP technology (MAGPIX, Millipore, Germany). This assay was performed with customized highly sensitive MILLIPLEX MAP Kit (Millipore, Germany) according to the manufacturer’s protocol.

### Enzyme-linked immune sorbent assay (ELISA)

Expressions of proinflammatory cytokines TNF-α, TGF-β, IL-2, and IL-1β in lung tissue and cell culture supernatants were assessed using commercially available ELISA kits (eBioscience, USA).

### Histopathological examination

The 5-micron lung tissue sections were stained with hematoxylin followed by eosin (H&E) and toluidine blue (TB) to observe pathological and morphological changes in the lung tissue. A semiquantitative histopathologic scoring was used to estimate the lung structural and cellular changes. The scoring was given as follows; (1) perivascular neutrophils (0—absent; 1—<10 per high-power field; 2—10–50 per high power field; 3—>50 per high-power field); (2) perivascular hemorrhage (0—absent; 1—patchy and mild; 2—extensive and mild; 3—extensive and marked); (3) neutrophilic margination in medium-sized vessels (0—absent; 1—present). A total inflammatory score (range, 0–7) was taken as the sum of the individual scores and quantified by a histopathologist who was unaware of treatment groups^20^. Mucosal mast cells were counted in 10 random fields per group as per the previous report^21^.

### Immunohistochemistry (IHC)

IHC was performed as per standard protocol reported earlier^22^. The sections were blocked with immune serum for avoiding non-specific binding. Then sections were incubated with anti-TNF-α, anti-NF-κB, and anti-HDAC-3 primary antibodies overnight at 4 °C. The further procedure was performed with the PolyExcel HRP/DAB Detection System kit (PathnSitu Biotechnologies, USA) and followed the manufacturer instructions. Then immune reactions were visualized by adding the DAB (3,3′-diaminobenzidine tetrachloride) and all sections were counterstained with hematoxylin. The protein expression was quantified by ImageJ Fiji.

### Western blot analysis

Whole cell, cytosolic, and nuclear protein isolation was performed as described earlier^23,24^. Protein concentration was estimated by BCA colorimetric assay kit as per manufacturer guidelines. Samples were loaded and subjected to SDS-PAGE. After electrophoresis, proteins were electrotransferred to nitrocellulose membrane (Bio-Rad, USA) and probed with primary and secondary antibodies before detecting with the enhanced chemiluminescence (ECL) solution (Bio-Rad, USA) by the chemdoc imaging system (Vilber Fusion Fx, France). The densitometric analysis was performed by ImageJ software, NIH, USA. β-Actin and histone H3 were used as internal controls for normalization of cytosolic as well as nuclear proteins.

### Real-time PCR

RNA was isolated from lung tissues using RNA isolation kit (Qiagen, Germany). After reverse transcription with Verso cDNA synthesis kit (Applied Biosystems, USA), real-time-PCR was performed on ABI 7500 system (Applied Biosystems, USA) using DyNAmoColourFlash SYBR Green qPCR kit (Thermo Fisher Scientific, USA) followed by the addition of forward and reverse primers (Integrated DNA Technologies, USA). After amplification, a melting-curve analysis was performed to verify the specificity of the reaction. The 18S rRNA gene was used as an internal control and results were determined by 2^−ΔΔCt^. Relative mRNA levels were expressed as the fold change over the normal control. The primer sequences were described in supplementary data (Supplementary Table S1).

### In silico molecular docking analysis

The crystal structure of the human TNF-α (PDB ID: 2AZ5)^25^ was used for molecular docking studies. For the ligand docking, the standard precision mode was selected. Docking was performed by using the standard protocol implemented in Maestro, version 9.7 and the ligands were docked against the active site of the targeted protein. No constraints were defined for the docking runs. The detailed procedure was described in supplementary data.

### In vitro macrophage bactericidal activity

Macrophagic bactericidal activity was performed as described previously with slight modifications^26^. RAW 264.7 cells alone or cells were treated with nimbolide (1 µM) and incubated in serum-free medium for 24 h. Then cells were stimulated with LPS (1 μg/ml) and incubated for 12 h. Later, cells were scraped in an ice-cold serum-free medium, washed twice with PBS and re-suspended in serum-free medium in triplicates. Wild-type *Pseudomonas aeruginosa* (Strain number: 424) (Pa) was procured from Microbial Type Culture Collection and Gene Bank, India. Bacteria from the exponential phase were added to the RAW 264.7 cells at a macrophage/bacteria ratio of 1:10. Bacterial phagocytosis was allowed to proceed for 60 min at 37 °C. At the end of the experiment, cells were lysed in 0.1% Triton X-100 solution and bacteria contained supernatant was collected and plated on to the nutrient-agar plates for colony counting to obtain bacterial uptake values. The Pa colonies were counted after 3 days of incubation at 37 °C. The macrophage bactericidal activity was calculated as follows.

$${\mathrm{\% }}\,{\mathrm{Pa}}\,{\mathrm{killed = }}\left( {{\mathrm{1 - }}\left[ {{\mathrm{Pa}}\,{\mathrm{C60/Pa}}\,{\mathrm{C0}}} \right]} \right) \times {\mathrm{100}}.$$

Here,

$${{\mathrm{Pa}}\,{\mathrm{C60 = Number}}\,{\mathrm{of}}\,{\mathrm{Pa}}\,{\mathrm{colonies}}\,{\mathrm{after}}\,{\mathrm{60}}\,{\mathrm{min}}\,{\mathrm{incubation}}{\mathrm{.}}}$$

$${{\mathrm{Pa}}\,{\mathrm{C0 = Number}}\,{\mathrm{of}}\,{\mathrm{Pa}}\,{\mathrm{colonies}}\,{\mathrm{at}}\,{\mathrm{the}}\,{\mathrm{beginning}}\,{\mathrm{of}}\,{\mathrm{incubation}}{\mathrm{.}}}$$

### Loss of TNF-α function and nuclear translocation of NF-κB and HDAC-3

BEAS-2B cells were transfected with TNF-α siRNA to knock down the TNF-α expression. Briefly, cells were seeded in 6-well plates at a density of 1 × 10^6^/well and cultured for 24 h at 37 °C. After attaining 80% confluency, cells were transfected with TNF-α and scrambled siRNA (50 nM) by Lipofectamine 3000 (Invitrogen, USA) according to the manufacturer instructions. In another set of experiment, cells were treated with nimbolide (2.5 µM). After 24 h of incubation, the cells were stimulated with 10 ng/ml of TNF-α for 30 min and determined the expression of TNF-α, NF-κB, and HDAC-3.

### Statistical analysis

Results were expressed as mean ± SEM, and *n* refers to the number of sample replicates. The statistical differences between the means were determined by one-way ANOVA followed by Tukey’s multiple comparison tests with Prism software (version 6.01; GraphPad, USA). p < 0.05 was considered to be statistically significant.

## Results

### Nimbolide reduces the LPS-induced nitrosative and oxidative stress

From the predicted physicochemical properties, it was found that nimbolide exhibited superior drug-likeness properties in terms of absorption, distribution, metabolism, and excretion-toxicity (ADME/T), hence it could be a potential lead molecule for treating various inflammatory diseases (Table S2**)**. In our experiment, cells (RAW 264.7, differentiated THP-1, MLE-12, A549, and BEAS-2B cells) were stimulated with LPS, further induced the oxidative stress and inflammatory signaling cascade. Initially, we performed MTT assay to ascertain the effect of nimbolide on the viability of aforementioned cell lines. Our results revealed that nimbolide had minimal effect on the viability in the tested cell lines up to 2.5 µM concentrations at 24 h post-treatment (Supplementary Figure S1A–E). Therefore, concentrations up to 2.5 µM were fixed to execute further molecular mechanistic studies.

**Fig. 1:**
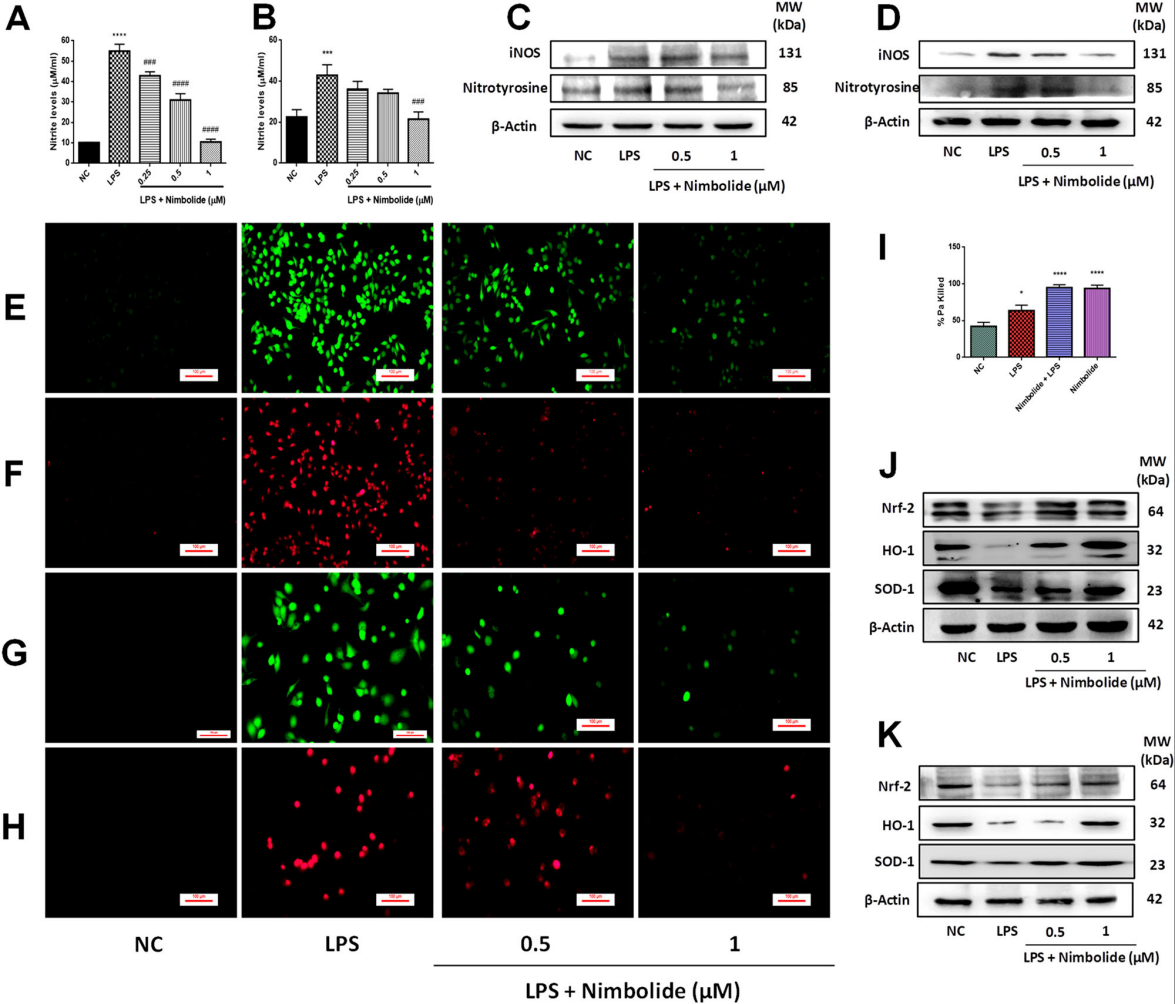
Nimbolide protects the lungs from nitrosative and oxidative stress. Cells were pre-treated with nimbolide for 24 h and oxidative stress was induced with LPS (1 µg/ml). After 24 h of incubation, the nitrite levels were measured in cell culture supernatants of (**a**) RAW 264.7 and (**b**) differentiated THP-1 cells by Griess assay. Whole cell protein was extracted and subjected to western blot and measured the expression of nitrotyrosine and iNOS in both (**c**) RAW 264.7 and (**d**) differentiated THP-1 cells. After 30 min of LPS stimulation, intracellular and mitochondrial ROS (mROS) levels were measured by (**e**, **g**) DCFDA and (**f**, **h**) MitoSOX Red staining in both RAW 264.7 and differentiated THP-1 cells, respectively. The fluorescent images were captured at ×200 magnification from different groups. (**i**) RAW264.7 cells were pre-treated with nimbolide (1 µM) and oxidative stress was induced by LPS (1 µg/ml). After 12 h of LPS exposure, cells were incubated with Pa and macrophage bactericidal activity was calculated by counting Pa colonies on nutrient agar plates. Western blot was performed to determine the Nrf-2, HO-1, and SOD1 expression in both (**j**) RAW 264.7 and (**k**) differentiated THP-1 cells from whole cell lysate. Data represented as mean ± SEM (*n* = 3 independent experiments). *P < 0.05, ***P < 0.001, and ****P < 0.0001 is significantly different from the normal control (NC) group; ^###^*P* < 0.001 and ^####^*P* < 0.0001 are significantly different from the LPS group

Among all nitric oxide synthases (NOSs), iNOS is mainly involved in the inflammatory diseases and further activates various inflammatory cytokines^27^. As nitrite is the final product of NO, these levels were found to be elevated in LPS stimulated group as compared to normal control (NC), moreover, these levels were significantly reduced by nimbolide in both RAW 264.7 (Fig. 1a) and differentiated THP-1 cells (Fig. 1b). The mechanism behind the downregulation of nitrite levels upon nimbolide treatment was determined by immunoblotting. The increased nitrotyrosine and iNOS proteins expression were observed with LPS stimulation. Furthermore, nimbolide significantly suppressed these nitrosative stress regulators in both RAW 264.7 (Fig. 1c and Supplementary Figure S2A, B and differentiated THP-1 cells (Fig. 1d and Supplementary Figure S2C, D) in a concentration- dependent manner. Similar results were found in mouse lung epithelial cells, where nimbolide inhibited the iNOS expression (Supplementary Figure S3A, B & D).

LPS induces the oxidative stress, which further activates the various inflammatory signaling pathways. Consistently, we observed that LPS induced both intracellular and mitochondrial ROS (mROS) levels, whereas nimbolide treatment (0.5 and 1 µM) significantly reduced the oxidative stress in both RAW 264.7 (Fig. 1e, f; Supplementary Figures S2E, F and S2I–L) and differentiated THP-1 cells (Fig. 1g, h; Supplementary Figures S2G, H and S2M-P). The mROS is considered to have a prime role in mediating bactericidal activity, even though nimbolide scavenged mROS but did not impair the bactericidal activity by murine macrophages, moreover, an enhanced antimicrobial activity was observed (Fig. 1i). The nuclear factor erythroid 2-related factor 2 (Nrf-2) manifests the antioxidant responsive genes that regulate the oxidative stress and maintain the cellular homeostasis through the induction of catabolism of superoxide ions by superoxide dismutase (SOD) and stress response protein, heme oxygenase-1 (HO-1)^28^. The protein expressions of Nrf-2, SOD1, and HO-1 were significantly enhanced by nimbolide in comparison with LPS control and reduces the oxidative stress observed in both RAW 264.7 (Fig. 1j and Supplementary Figure S2Q–S) and differentiated THP-1 cells (Fig. 1k and Supplementary Figure S2T–V). Similar results were observed in MLE-12 cells, where nimbolide enhances the Nrf-2 expression (Supplementary Figure S3A, C & E)

### Nimbolide normalizes the LPS-induced physiological changes and downregulates the inflammation responsive cells in bronchoalveolar lavage fluid (BALF) and blood

**Fig. 2:**
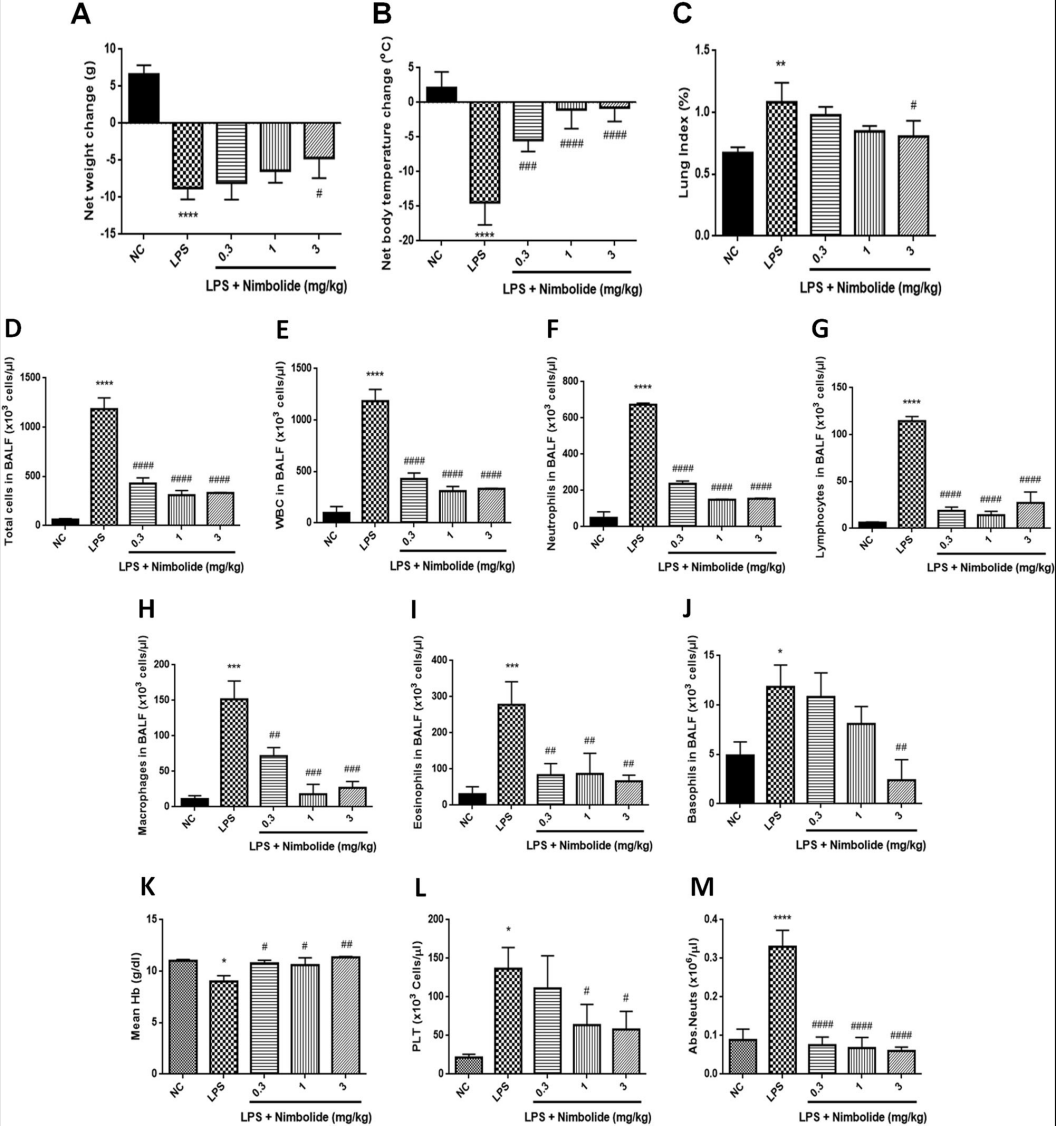
Nimbolide restores LPS-induced physiological, bronchoalveolar lavage (BAL) cytological and hematological changes. The changes in (**a**) body weight, (**b**) temperature and (**c**) percentage lung index were determined 12 h after LPS challenge. BALF was collected 12 h after LPS administration to measure the number of (**d**) total cells, (**e**) WBC, (**f**) neutrophils, (**g**) lymphocytes, (**h**) macrophages, (**i**) eosinophils and (**j**) basophils. Whole blood was collected and analyzed for (**k**) mean hemoglobin (Hb), (**l**) platelets (PLT), and (**m**) absolute neutrophil count after LPS induction. Data presented as mean ± SEM (*n* = 8 animals per group). *P < 0.05, **P < 0.01, ***P < 0.001, and ****P < 0.0001 are significantly different from the NC group; ^#^*P* < 0.05, ^##^*P* < 0.01, ^###^*P* < 0.001, and ^####^*P* < 0.0001 are significantly different from the LPS group

LPS altered the normal physiology with severe pathological changes such as hypothermia and weight loss at 12 h of post exposure. In addition, LPS induced the accumulation of serous fluids in the lungs which resulted in pulmonary edema evident with increased lung weight index. However, nimbolide treatment at 3 mg/kg concentration did not impair the body weights but reduced the abnormal lung weight index. Whereas hypothermia was normalized significantly at all the doses tested (0.3, 1 and 3 mg/kg) (Fig. 2a–c). Previous reports suggest that LPS elevates total cells, white blood cells (WBC), neutrophils, lymphocytes, macrophages, eosinophils and basophils in BALF, which play a key role in propagating inflammation^29^. Whereas, nimbolide treatment significantly suppressed these cell counts in BALF (Fig. 2d–j**)**.

Similarly, in whole blood analysis, it was found that LPS induced the platelets and absolute neutrophils count along with decreased hemoglobin (Hb) levels, whereas nimbolide intervention did not alter these levels (Fig. 2k–m).

### Nimbolide reduces LPS-induced pathological consequences and mast cell infiltration in lung tissues

**Fig. 3:**
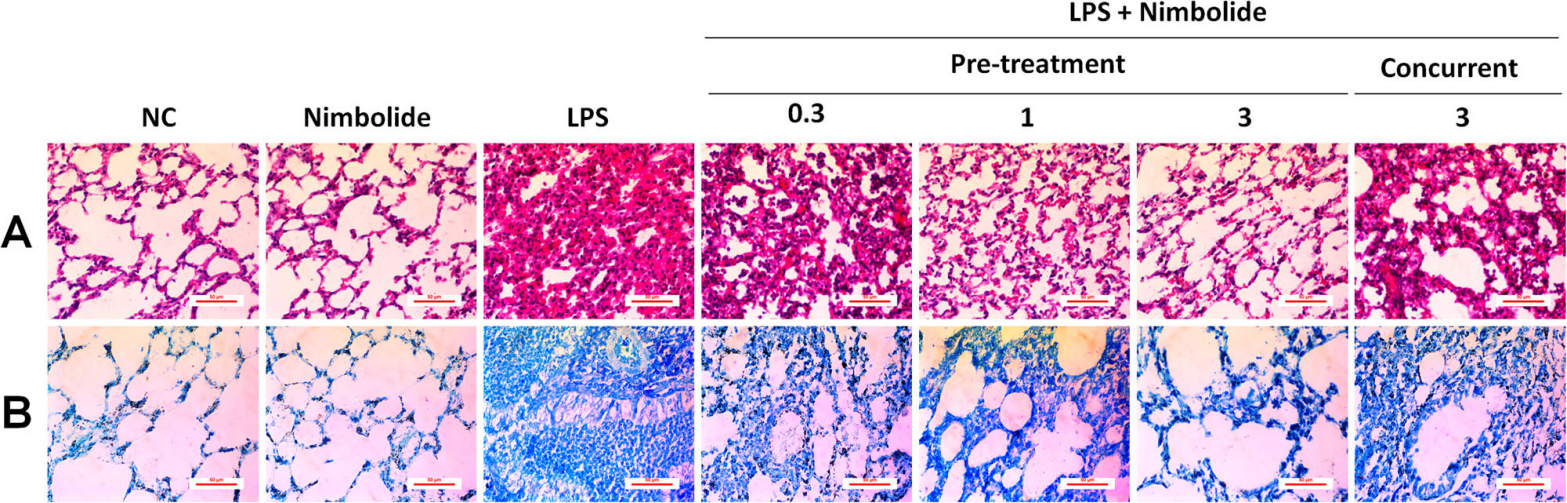
Nimbolide reverses lung injury and pulmonary edema in LPS challenged mice. Lungs from different groups were collected and sections were prepared for pathological examination. Histopathological studies were performed with (**a**) hematoxylin and eosin (H&E) and (**b**) toluidine blue (TB) staining. The images were captured by a bright field microscope at ×400 magnification

LPS instilled animal group exhibited severe pathological changes and caused the endothelial barrier dysfunction. As evident from H&E results, it was observed that inflammatory cells such as neutrophils influx into the alveolar spaces, thus led to the thickening of an interalveolar septum (Fig. 3a and Supplementary Figure S4A). Additionally, from TB staining, it was observed that there was a dramatic accumulation and infiltration of a mast cell density (Fig. 3b and Supplementary Figure S4B). Moreover, nimbolide pre-treatment remarkably repressed both pathological consequences at 1 and 3 mg/kg doses. Whereas in concurrent administration at 3 mg/kg, there was no significant reduction observed in comparison to LPS instilled group. However, when nimbolide alone treatment at 3 mg/kg was tested, we did not observe the alveolar structural changes and appeared normal. Hence, further experiments were performed in pre-treatment groups of nimbolide to investigate its molecular mechanism.

### Repression of inflammatory signaling responsive cytokines and chemokines by nimbolide in LPS-induced lung injury

**Fig. 4:**
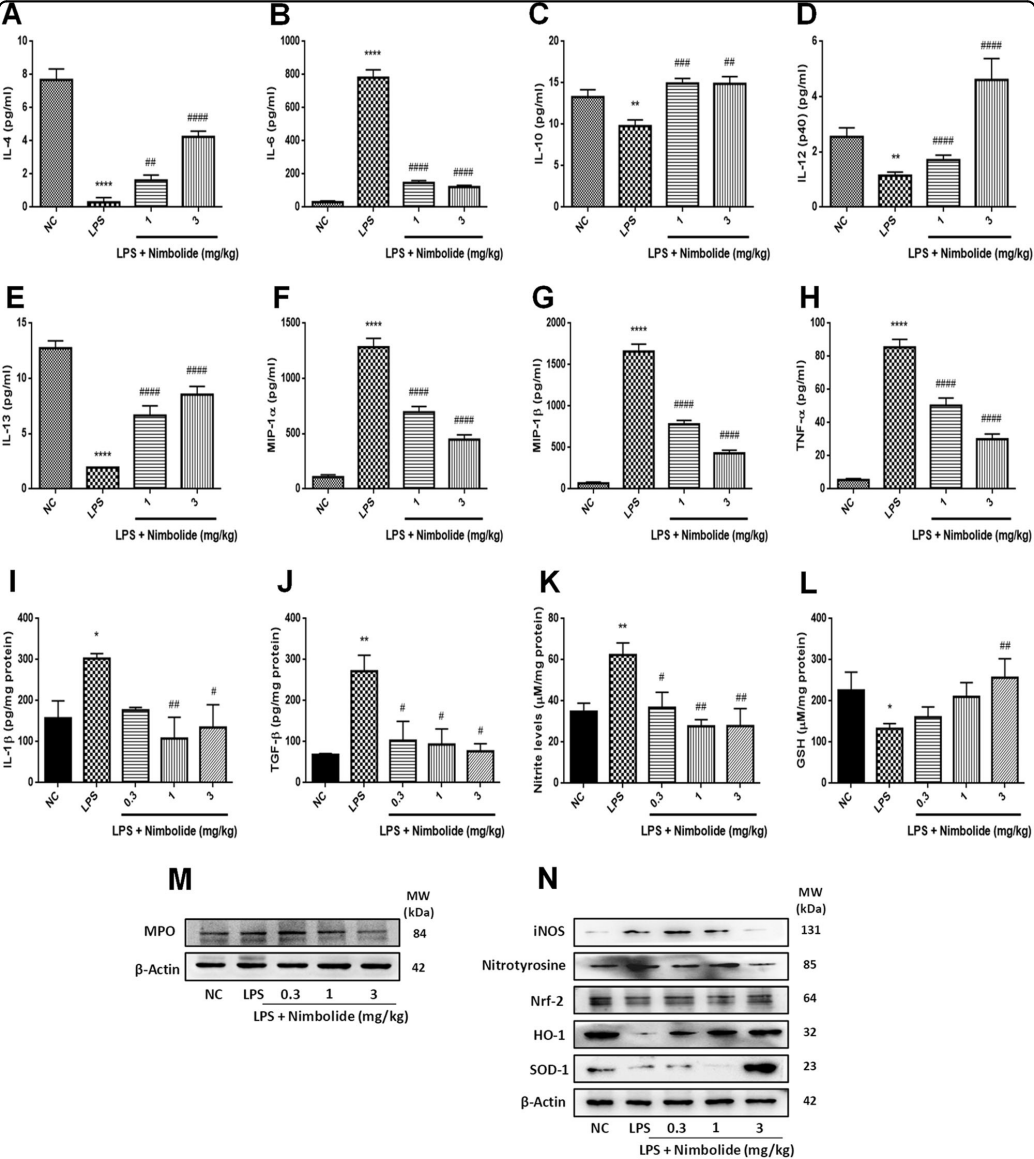
Nimbolide modulates the pro- and anti-inflammatory cytokines and chemokines. The proinflammatory cytokines and chemokines (IL-6, IL-12 (p40), MIP-1α, MIP-1β, and TNF-α) and anti-inflammatory cytokines (IL-4, IL-10, and IL-13) expression were determined by multiplex and ELISA. **a**–**h** Animal lung tissue lysate was prepared and evaluated for the levels of aforementioned inflammatory cytokines and chemokines by multiplex. **i**, **j** lung tissue supernatants were subjected to ELISA to determine IL-1β and TGF-β cytokines expression. **k** Griess assay was performed to determine the nitrite levels in cell lysate of lung tissues. **l** The antioxidant GSH levels were measured in lung tissue. **m** The MPO as well as (**n**) iNOS, nitrotyrosine, Nrf-2, HO-1, and SOD-1 protein expressions were determined by immunoblot analysis. Data presented as mean ± SEM (n=8 mice per group). *P < 0.05, **P < 0.01, and ****P < 0.0001 are significantly different from the NC group; ^#^*P* < 0.05, ^##^*P* < 0.01, ^###^*P* < 0.001, and ^####^*P* < 0.0001 are significantly different from the LPS group

We performed multiplex analysis to determine the cytokine and chemokine levels. We observed elevated proinflammatory cytokines such as IL-1β, IL-6, IL-12 (p40), TNF-α, TGF-β, and chemokines (MIP-1α and MIP-1β) with reduced levels of anti-inflammatory cytokines including IL-4, IL-10, and IL-13 were observed in LPS treated groups^30^. Furthermore, nimbolide significantly reduced the proinflammatory cytokines and chemokines, concomitantly induced the anti-inflammatory cytokines in lung tissues (Fig. 4a–h), respectively. To further confirm with the multiplex results, we performed ELISA to investigate the expression of cytokines including IL-1β and TGF-β in lung tissues (Fig. 4i, j), where LPS driven cytokine levels were significantly reduced by nimbolide which is consistent with multiplex results. Additionally, these cytokines and chemokine levels were analyzed in RAW 264.7 and A549 cells, where nimbolide pre-treatment significantly inhibited the cytokine-mediated inflammation (Supplementary Figure S5). The anti-inflammatory activity of nimbolide was mediated by suppressing the LPS-induced nitrite levels with a simultaneous increase in GSH levels (Fig. 4k, l**)**. Moreover, LPS induces the oxidative and nitrosative stress via myeloperoxidase (MPO), iNOS and nitrotyrosine, which was significantly decreased by nimbolide through the upregulation of antioxidative regulators such as Nrf-2, SOD-1, and HO-1 expression (Fig. 4m, n and Supplementary Figure S6a–f).

### Nimbolide inhibits TNF-α mediated pulmonary inflammation

**Fig. 5:**
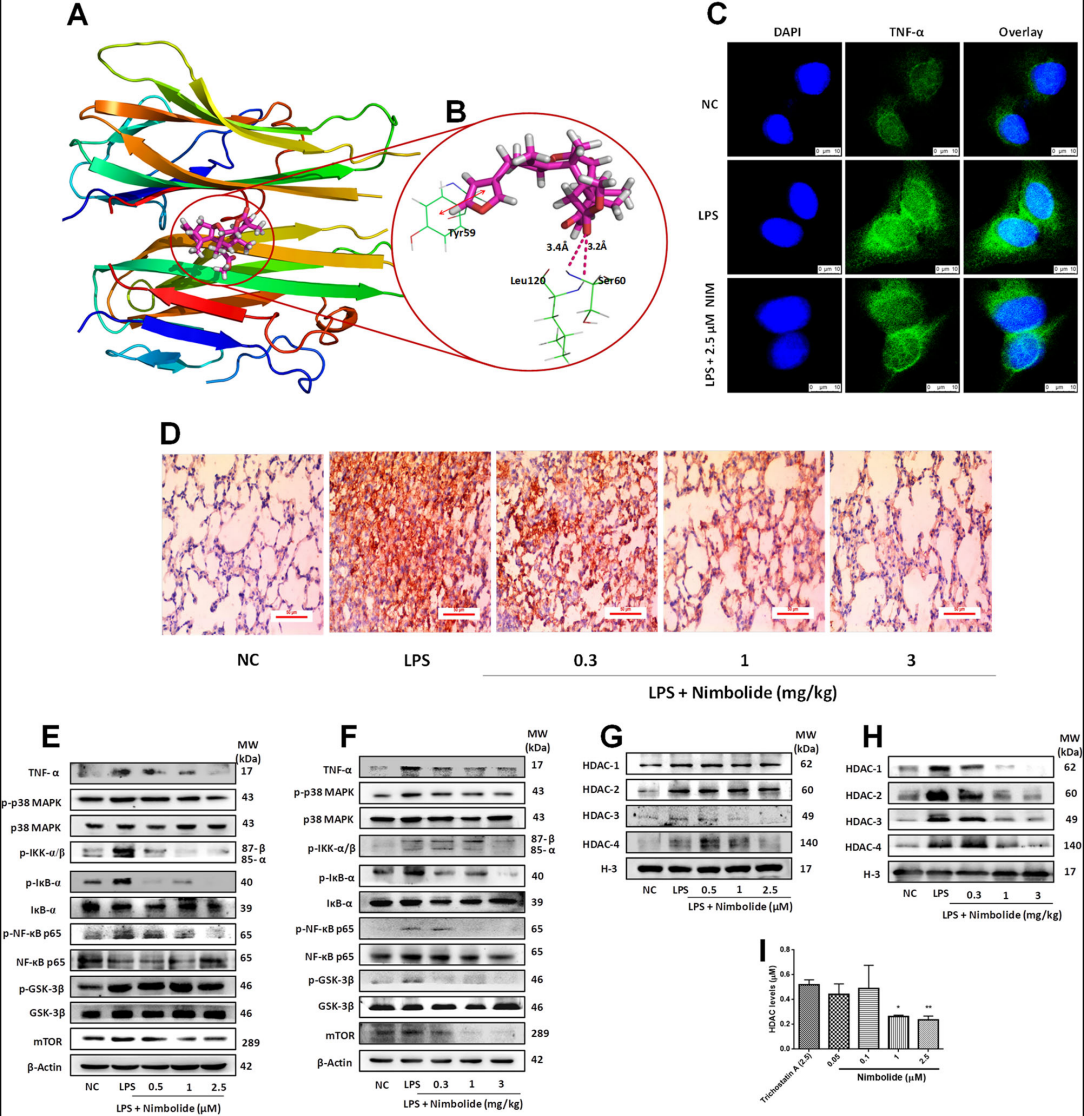
Nimbolide interacts with TNF-α and inhibits TNF-α regulated inflammatory signaling. **a** Docking model of nimbolide in the active site of TNF-α (PDB ID: 2AZ5) and (**b**) its ligand-protein interactions in the binding site of TNF-α. The dark pink dashed lines represent hydrogen bonds. H-bond distances (in Å) between heteroatoms of ligand and amino acid residues are as follows: Ser60 (3.4Å) and Leu120 (3.2Å). The red line indicates arene-arene interaction with Tyr59. A549 cells were pre-treated with nimbolide for 24 h and stimulated by LPS (1 µg/ml) for 12 h. Animals were pre-treated with nimbolide (0.3, 1 and 3 mg/kg) for 5 days later LPS (50 μg) was administered. **c** TNF-α protein expression was analyzed by confocal microscope. **d** A 5-µm-sized sections of lung tissues were subjected to IHC to determine TNF-α expression and images were captured at ×400 magnification. Protein expressions of TNF-α, p-p38 MAPK, p-IKK-α/β, p-IκB-α, p-NF-κB, p-GSK-3β, and mTOR were determined by western blotting in (**e**) A549 cells and (**f**) lung tissues, respectively. The HDACs such as HDAC-1, 2, 3, and 4 protein expressions were studied in (**g**) A549 cells and (**h**) lung tissues. **i** Nimbolide (0.05, 0.1, 1, and 2.5 µM) upon HDAC levels were analyzed by HDAC fluorometric kit and HDAC inhibitory activity was compared with trichostatin A (2.5 µM). Data represented as mean ± SEM (n=3 independent experiments). *P < 0.05 and **P < 0.01 are significantly different from the trichostatin A group

As evident from ELISA and multiplex results, nimbolide was found to have a key role in inhibiting TNF-α. These results prompted us to decipher the molecular interactions of nimbolide with TNF-α, hence docking studies were performed. The results of molecular docking along with hydrogen bonding as well as hydrophobic interactions of ligands with TNF-α were depicted in Table S3. The docking results illustrated the predicted binding modes and the detailed protein inhibitor interactions of nimbolide with TNF-α. From the molecular docking analysis, it was observed that nimbolide established two hydrogen bonds between the methyl ester group of the compound and the active site residues (Leu120 and Ser60) of TNF-α. Additionally, the furan ring of the compound formed a *π*–*π* stacking interaction (arene-arene interaction) with Tyr59. Furthermore, several hydrophobic interactions were found between nimbolide and active site residues, e.g., Leu57, Tyr59, Tyr119, Leu120, Val123, Tyr151, and Ile155 are the other residues that stabilized the binding of nimbolide in the active site of TNF-α. The binding model of nimbolide also revealed that they share one common hydrogen bonding and arene-arene interactions with the key residues of the active site as shown by co-crystallized ligand. Moreover, docked nimbolide and co-crystallized ligand suggested that nimbolide also occupies the binding pocket in a similar fashion to that of co-crystallized ligand (Fig. 5a, b). To further affirm these results, we extensively analyzed the effect of nimbolide on TNF-α expression. Collectively, the confocal (Fig. 5c) and IHC (Fig. 5d and Supplementary Figure S7A) results unveiled that nimbolide significantly decreased the LPS-induced TNF-α expression at translational level.

We further investigated the role of nimbolide on TNF-α mediated downstream inflammatory signaling pathways, where nimbolide markedly inhibited the LPS-induced phosphorylation of TNF-α regulated p38 MAPK, IKK-α/β, IκB-α, p65 NF-κB, GSK-3β along with mTOR expression in A549 cells (Fig. 5e and Supplementary Figure S7B–H) as well as lung tissues (Fig. 5f and Supplementary Figure S7I–O). Further, it is our interest to explore the effect of nimbolide on epigenetic alterations which are commonly associated with LPS in inflammatory conditions. Here, we found aberrant expression of HDAC-1, 2, 3, and 4 upon LPS stimulation. Interestingly, nimbolide repressed the HDACs expression in a dose-dependent manner in vitro (A549 cells; Fig. 5g and Supplementary Figure S7P–S) and the results were consistent with in vivo (Fig. 5h and Supplementary Figure S7T–W). To confirm the HDAC inhibitory activity of nimbolide, we performed a fluorimetric based HDAC assay. Where, we found that there was a significant reduction in HDAC levels by nimbolide at different concentrations such as 0.05, 0.1, 1, and 2.5 µM, where the HDAC levels were 0.44 ± 0.06, 0.49 ± 0.13, 0.26 ± 0.03, and 0.24 ± 0.01 µM, respectively in comparison with trichostatin A, which inhibited the HDAC levels up to 0.52 ± 0.02 µM at 2.5 µM concentration (Fig. 5i).

### Nimbolide inhibits NF-κB and HDAC-3 nuclear translocation

**Fig. 6:**
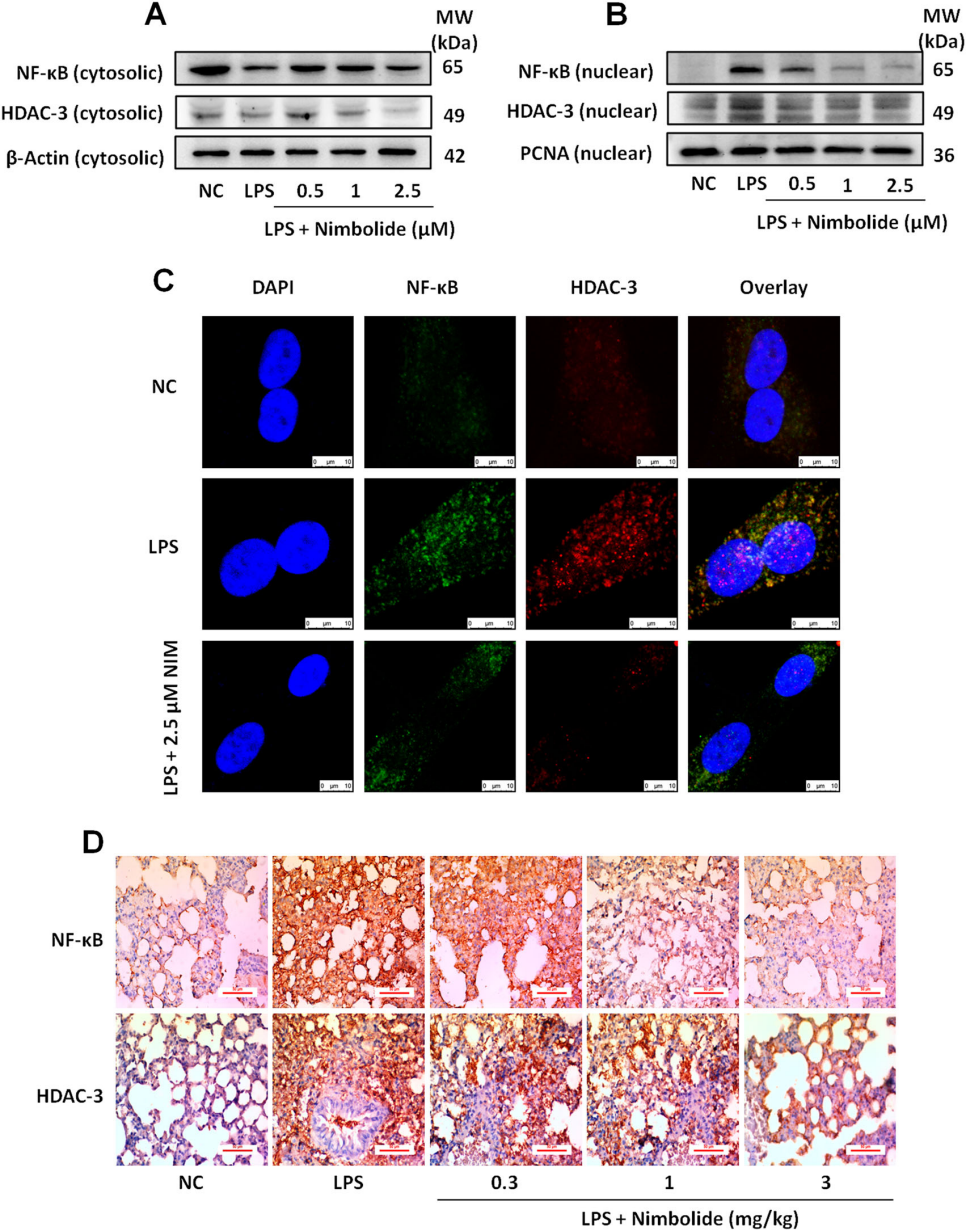
Nimbolide suppresses NF-κB and HDAC-3 protein expression and nuclear translocation. A549 cells were pre-treated with nimbolide for 24 h. After 30 min of LPS (1 µg/ml) incubation, both cytosolic and nuclear proteins were isolated. NF-κB and HDAC-3 protein levels were observed in (**a**) cytosolic and (**b**) nuclear fraction of A549 cells. **c** Similarly, the protein expression of NF-κB and HDAC-3 was analyzed by confocal microscope in A549 cells. **d** Microsections of lung tissues were subjected to IHC to determine NF-κB and HDAC-3 expressions and images were captured at ×400 magnification

The western blot analysis results inferred that LPS stimulation significantly enhanced the translocation of NF-κB and HDAC-3 into the nucleus, whereas nimbolide treatment hindered the nuclear translocation, where NF-κB and HDAC-3 were confined to the cytoplasm (Fig. 6a, b and Supplementary Figure S8A–D**)**. For further confirmation of nimbolide inhibitory mechanism of nuclear translocation, we performed IF to examine the effect of nimbolide on NF-κB and HDAC-3 expression. Here, nimbolide significantly inhibited the NF-κB and HDAC-3 expression and co-localization in contrast to LPS treatment in A549 cells (Fig. 6c). The similar pattern of decreased expression was observed by IHC, where LPS surged the NF-κB and HDAC-3 protein expression. Further treatment with nimbolide repressed the protein expression by truncating the immunopositivity against LPS-induced alveolar inflammation (Fig. 6d and Supplementary Figure S8E, F). Additionally, nimbolide significantly attenuated the LPS-induced TNF-α, NF-κB, and HDAC-3 mRNA levels at transcriptional level studied by RT-PCR (Supplementary Figure S9).

**Fig. 7:**
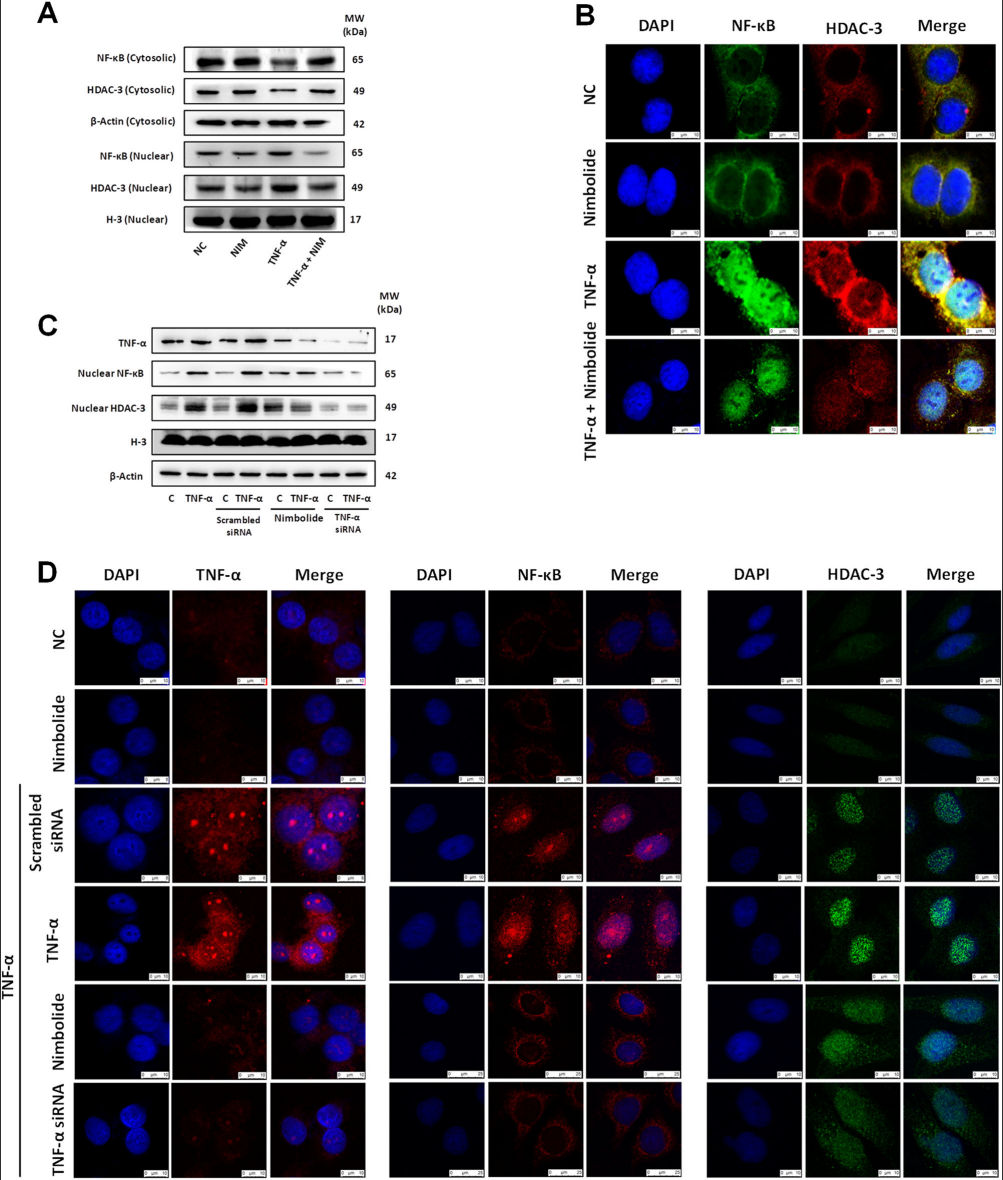
Nimbolide inhibits TNF-α regulated NF-κB and HDAC-3 protein expression and nuclear translocation. A549 cells were treated with nimbolide (2.5 µM) for 24 h and stimulated with TNF-α (10 ng/ml) for 30 min. **a** NF-κB and HDAC-3 protein levels were observed in both cytosolic and nuclear fraction of A549 cells by western blotting. **b** The confocal analysis was performed to determine the protein expressions of NF-κB and HDAC-3. All the images were captured at ×400 magnification. BEAS-2B cells were transfected with TNF-α and scrambled siRNA and incubated for 24 h. In another set of the experimental group, cells were pre-treated with nimbolide (2.5 µM) for 24 h. Then, cells were stimulated with TNF-α (10 ng/ml) for 30 min except for nimbolide alone (NIM) group (2.5 µM). The expression of TNF-α, NF-κB and HDAC-3 levels were measured by (**c**) western blotting and (**d**) confocal analysis. The images were captured at ×400 magnification

Consistent with LPS activity, we observed TNF-α induced the nuclear translocation of NF-κB and HDAC-3, whereas nimbolide significantly reduced this effect in A549 cells confirmed by western blotting (Fig. 7a and Supplementary Figure S10A–D) and IF analysis (Fig. 7b). Additionally, we evaluated the role of nimbolide in TNF-α-mediated NF-κB and HDAC-3 nuclear translocation in bronchial epithelial cells. TNF-α expression was silenced by siRNA and nuclear NF-κB and HDAC-3 levels were examined, where TNF-α siRNA treated groups did not exhibit the nuclear translocation of NF-κB and HDAC-3, which further confirms that TNF-α is essential for activating the inflammatory cascade through nuclear translocation in ARDS. Moreover, nimbolide significantly reduced the nuclear translocation and showed similar results as TNF-α siRNA treated group, which was confirmed by both immunoblotting (Fig. 7c and Supplementary Figure S10E–G) and immunofluorescence (Fig. 7d). However, the nimbolide alone did not show any significant changes in the expression of TNF-α, nuclear NF-κB, and HDAC-3 levels as compared to LPS group and appeared as normal (Supplementary Figure S11). Moreover, the endotoxin-mediated nitrosative stress regulators were unaltered by nimbolide alone and showed similar results as NC (Supplementary Figure S12).

## Discussion

ARDS is a life-threatening condition, which is predominantly associated with a massive number of inflammatory cells migrating to the lung, which could lead to the release of inflammatory mediators that disrupts the alveolar capillary epithelial and endothelial barrier^31^. This loss of integrity increases the permeability and exudates protein-rich serous fluids, which finally leads to lung edema. For this critical anomaly, the corticosteroids would be the preferred choice for treating ARDS patients due to its potent anti-inflammatory and anti-fibrotic activity, but these agents have numerous adverse effects, thus limit the use. To overcome, nowadays, researchers are looking for novel and safer therapeutic approaches for treating ARDS symptoms^32^. Nimbolide, a natural terpenoid lactone is widely explored in the treatment of various acute and chronic inflammatory diseases^33^. As in terms of toxicity, previous reports suggest that nimbolide was found to be toxic upon i.p. administration with LD_50_ of 225 mg/kg body weight in adult male mice^34^. In the present study, we have administered much safer doses (0.3, 1, and 3 mg/kg), this sheds the importance of nimbolide in pharmacotherapy. Intriguingly, with the in silico predicted studies, it was observed that nimbolide possess significant physicochemical properties and has the potential to exhibit the properties of drug likeness. The diverse effects of nimbolide and novel insights into its molecular mechanism have been unveiled in the present study.

Enhanced ROS levels activate multiple inflammatory signaling pathways which contribute to pulmonary inflammation. Consistent with literature, we observed an increase in intracellular and mROS levels by LPS in both cultured macrophages and lung tissues. The protective effect of nimbolide was attributed through the decrease in intracellular and mROS production. In accordance, nimbolide modulated a variety of downstream responses that are typically associated with ROS production, apparently through the induction of GSH levels^35^ and upregulation of Nrf-2, SOD1, and HO-1 expression^28^. In addition, nimbolide markedly suppressed MPO expression, which is a key regulator of oxidative stress^36^, thus maintained the redox homeostasis. TNF-α activation by LPS in macrophages induces the mROS levels, thus initiates antimicrobial response^37^. In our study, we noticed that nimbolide reduced mROS levels with antioxidant activity, without any impairment in bactericidal activity. In turn, an enhanced bactericidal response by macrophages was observed as compared to the control with unknown mechanism. These results were consistent with Hidalgo HA et al., where dexamethasone exhibited TNF-α inhibitory mechanism, without impairing the macrophage bactericidal activity against Pa^26^. NO is a well-known nitrosative stress inducer, which is produced by iNOS accompanied by nitrotyrosine and plays a crucial role in ARDS pathology^38,39^. Here, in our study, we found that nimbolide sequestered the nitrite levels by inhibiting NT and iNOS expression thus, attenuated the exaggerated RNS.

The inflammatory cells such as dendritic cells, macrophages, neutrophils, lymphocytes and eosinophils disrupt the endothelial barrier^40–42^. Our studies demonstrated that LPS profoundly elevated myriad of inflammatory cells such as total cells, mast cells, WBC, neutrophils, lymphocytes, macrophages, eosinophils, and basophils in BALF^43^. Strikingly, these initiating aforementioned cytological parameters were reduced markedly with nimbolide treatment. Additionally, LPS induced the platelets and neutrophil count, with concomitantly reduction in Hb levels, whereas nimbolide did not alter these levels.

LPS induced pulmonary inflammation is associated with body temperature alterations like hypothermia, increased lung index due to the inflammatory cell infiltration which could lead to pleural edema^20^. We observed increased expression of inflammatory cytokines such as IL-1β, IL-2, IL-6, IL-12 (p40), TNF-α, and TGF-β as well as chemokines MIP-1α and MIP-1β in LPS instilled group, further their levels were effectively attenuated by nimbolide treatment. The anti-inflammatory cytokines such as IL-4, IL-10, and IL-13 are expressed by T helper cells 2 and involve in the inhibition of LPS-induced proinflammatory cytokine synthesis^44^. In the present study, nimbolide upregulated the expression of IL-4, IL-10, and IL-13 and preserved the lungs alveolar structure. On the other hand, nimbolide treatment significantly normalized the body weights and exhibited normothermia.

TNF-α has been implicated as a key cytokine in ARDS, which is produced by various cell types including epithelium, endothelium and activated macrophages in response to inflammatory stimuli and considered to be the “master regulator” of proinflammatory cytokines production^45^. TNF-α acts as a central player in initiation and perpetuation of inflammation by orchestrating inflammatory cells activation and recruitment^46,47^. Hence, pharmacological agents that can either suppress the production of TNF-α or block its biological actions may have potential therapeutic value. Consistently, in the present study, aberrant TNF-α expression was observed in response to LPS stimulation, evidenced at both transcriptional and translational level. It is worthwhile to mention that among the cytokines, a dramatic decline in TNF-α have been observed with nimbolide treatment. On the basis of these results, we speculated that nimbolide executed anti-inflammatory effects through the suppression of TNF-α, which could be the key target. To validate our prediction, we next performed molecular modeling, as hypothesized there was a striking correlation, where we found that nimbolide molecule strongly interacted with TNF-α as compared to its existed co-crystal. Hence, these results prompted us to determine further underlying molecular events associated with TNF-α.

Previous studies have demonstrated a significant role of TNF-α in NF-κB signaling pathway^48^. Where, IKK-α/β is activated by TNF-α, further it causes phosphorylation of IκB, which leads to p65 NF-κB nuclear translocation and induces inflammatory cytokine gene expression^49^. The activation of NF-κB is critical in mediating in the development of endotoxin-induced ARDS, owing to the worse outcome^50^. Moreover, accumulating evidence suggested that the inflammatory regulator, IκB-α concomitantly induces HDAC-3 nuclear translocation and further regulates inflammation^51^. Intriguingly, H Zhu et al. reported that activation of HDAC-3 and mROS induce the TNF-α expression as a result of crosstalk^52^.

To gain insight into the nimbolide mechanism, epigenetic alterations of HDAC’s have been studied. Furthermore, immunoblot results corroborated that LPS stimulation increased HDAC-1, 2, 3, and 4, where nimbolide effectively reduced the protein expression dose-dependently. These results were consistent with HDAC enzyme assay, where nimbolide inhibited HDAC levels significantly as compared to trichostatin-A. Thus, nimbolide could be the potential emerging molecule as multiple HDAC inhibitor.

In line with the literature, to delineate the crosstalk mechanism, immunoblotting and confocal analysis were performed in A549 cells with LPS/TNF-α stimulation, where we found TNF-α regulated IκB mediated NF-κB and HDAC-3 translocation into the nucleus. For more specificity, bronchial epithelial cells were stimulated with TNF-α and observed the consistent mechanism. Interestingly, when we knocked out the TNF-α expression, disappearance of NF-κB and HDAC-3 was observed in the nucleus. Similarly, nimbolide treatment led to the effective disruption of LPS/TNF-α mediated NF-κB and HDAC-3 translocation into the nucleus, by inhibiting the phosphorylation of IKK-α/β and IκB-α, and these results were comparable with specific TNF-α loss of function. Also, we experimentally found that nimbolide remarkably inhibited other TNF-α mediated downstream signaling pathways including, p38 MAPK, GSK-3β, and mTOR expression.

**Fig. 8:**
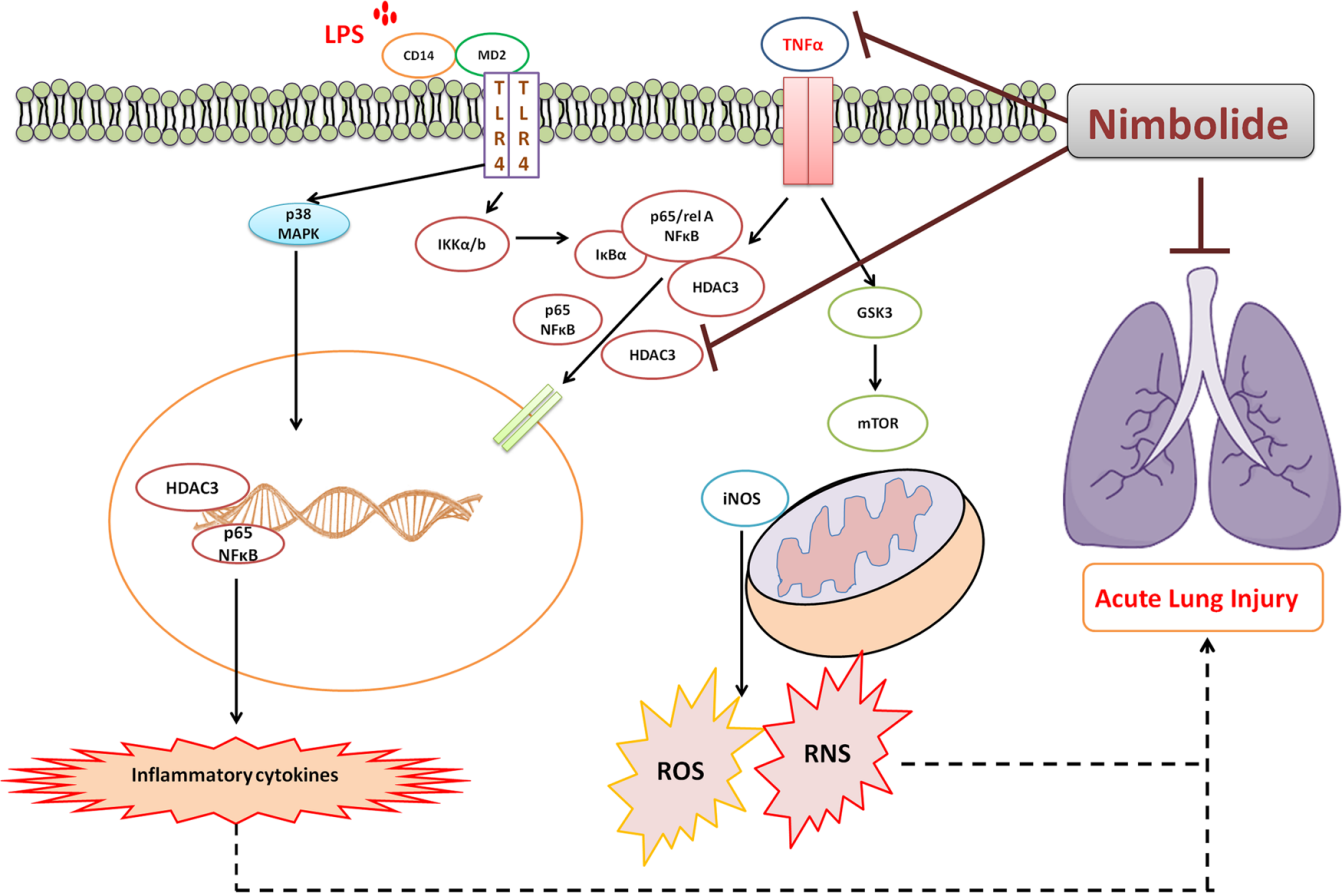
Amelioration of LPS-induced alveolar inflammation by nimbolide. A schematic diagram represents the molecular mechanism of nimbolide in LPS-induced ARDS. The bacterial endotoxin, lipopolysaccharide (LPS) binds to its cognate toll-like receptor 4 (TLR-4) and the co-receptor cluster of differentiation 14 (CD14), which results in neutrophil accumulation, elevated vascular permeability, provocation of pulmonary edema. Nimbolide suppresses the nuclear translocation of NF-κB and HDAC-3. RNS- and ROS-induced ARDS is prevented by nimbolide by different canonical pathways

Our data provided a potential mechanistic link between the TNF-α or LPS induced nuclear translocation of NF-κB and HDAC-3. A schematic representation of the protection of nimbolide from LPS-induced ARDS inflammatory symptoms is illustrated in Fig. 8. This study emphasized the potential role of nimbolide in inhibiting NF-κB and HDAC-3 translocation, thereby reducing inflammatory cytokines and maintaining redox balance, thus alleviate the inflammatory symptoms associated with ARDS conditions.

## Supplementary information

Supplementary data

## References

[1] Johnson ER, Matthay MA (2010). Acute Lung Injury: epidemiology, pathogenesis, and treatment. J. Aerosol Med. Pulm. Drug. Deliv..

[2] Rubenfeld GD (2005). Incidence and outcomes of acute lung injury. N. Engl. J. Med..

[3] Li C, Bo L, Liu W, Lu X, Jin F (2015). Enteral immunomodulatory diet (omega-3 fatty acid, γ-linolenic acid and antioxidant supplementation) for acute lung injury and acute respiratory distress syndrome: an updated systematic review and meta-analysis. Nutrients.

[4] Jeyaseelan S, Chu HW, Young SK, Freeman MW, Worthen GS (2005). Distinct roles of pattern recognition receptors CD14 and toll-like receptor 4 in acute lung injury. Infect. Immun..

[5] Zanoni I (2011). CD14 controls the LPS-induced endocytosis of toll-like receptor 4. Cell.

[6] Ramana KV (2006). Aldose reductase mediates the lipopolysaccharide-induced release of inflammatory mediators in RAW264.7 murine macrophages. J. Biol. Chem..

[7] Pushpakumar S (2017). Toll-like receptor 4 deficiency reduces oxidative stress and macrophage mediated inflammation in hypertensive kidney. Sci. Rep..

[8] Mukhopadhyay S, Hoidal JR, Mukherjee TK (2006). Role of TNFalpha in pulmonary pathophysiology. Respir. Res..

[9] Ashburner BP, Westerheide SD, Baldwin AS (2001). The p65 (RelA) subunit of NF-kappaB interacts with the histone deacetylase (HDAC) corepressors HDAC1 and HDAC2 to negatively regulate gene expression. Mol. Cell. Biol..

[10] Gonneaud A, Gagné JM, Turgeon N, Asselin C (2014). The histone deacetylase Hdac1 regulates inflammatory signalling in intestinal epithelial cells. J. Inflamm. Lond. Engl..

[11] Ziesché E (2013). The coactivator role of histone deacetylase 3 in IL-1-signaling involves deacetylation of p65 NF-κB. Nucleic Acids Res..

[12] Leus NGJ (2016). HDAC 3-selective inhibitor RGFP966 demonstrates anti-inflammatory properties in RAW 264.7 macrophages and mouse precision-cut lung slices by attenuating NF-κB p65 transcriptional activity. Biochem. Pharmacol..

[13] Usui T (2012). HDAC4 mediates development of hypertension via vascular inflammation in spontaneous hypertensive rats. Am. J. Physiol. Heart Circ. Physiol..

[14] Dong W (2017). Sodium butyrate activates NRF2 to ameliorate diabetic nephropathy possibly via inhibition of HDAC. J. Endocrinol..

[15] Leus NGJ (2017). HDAC1-3 inhibitor MS-275 enhances *IL10* expression in RAW264.7 macrophages and reduces cigarette smoke-induced airway inflammation in mice. Sci. Rep..

[16] Pooladanda V, Bandi S, Mondi SR, Gottumukkala KM, Godugu C (2018). Nimbolide epigenetically regulates autophagy and apoptosis in breast cancer. Toxicol. Vitr..

[17] Nekkanti S (2017). Synthesis of 1,2,3-triazolo-fused-tetrahydro-β-carboline derivatives via 1,3-dipolar cycloaddition reaction: cytotoxicity evaluation and DNA-binding studies. ChemistrySelect.

[18] Jain A (2017). Liposphere mediated topical delivery of thymoquinone in the treatment of psoriasis. Nanomed. Nanotechnol. Biol. Med..

[19] Rahman I, Kode A, Biswas SK (2006). Assay for quantitative determination of glutathione and glutathione disulfide levels using enzymatic recycling method. Nat. Protoc..

[20] Card JW (2006). Gender differences in murine airway responsiveness and lipopolysaccharide-induced inflammation. J. Immunol..

[21] Pohl CS (2017). Early weaning stress induces chronic functional diarrhea, intestinal barrier defects, and increased mast cell activity in a porcine model of early life adversity. Neurogastroenterol. Motil..

[22] Godugu C (2013). Inhalation delivery of telmisartan enhances intratumoral distribution of nanoparticles in lung cancer models. J. Control. Release.

[23] Godugu C, Patel AR, Doddapaneni R, Somagoni J, Singh M (2014). Approaches to improve the oral bioavailability and effects of novel anticancer drugs berberine and betulinic acid. PLoS ONE.

[24] Kaur J, Tikoo K (2013). p300/CBP dependent hyperacetylation of histone potentiates anticancer activity of gefitinib nanoparticles. Biochim. Biophys. Acta.

[25] He MM (2005). Small-molecule inhibition of TNF-alpha. Science.

[26] Hidalgo HA, Helmke RJ, German VF, Mangos JA (1992). The effects of cyclosporine and dexamethasone on an alveolar macrophage cell line (NR8383). Transplantation.

[27] Speyer CL (2003). Regulatory effects of iNOS on acute lung inflammatory responses in Mice. Am. J. Pathol..

[28] Ma Q (2013). Role of Nrf2 in oxidative stress and toxicity. Annu. Rev. Pharmacol. Toxicol..

[29] Whitehead GS (2017). TNF is required for TLR ligand-mediated but not protease-mediated allergic airway inflammation. J. Clin. Invest..

[30] Cross LJM, Matthay MA (2011). Biomarkers in acute lung injury: insights into the pathogenesis of acute lung injury. Crit. Care Clin..

[31] Wilson KC, Saukkonen JJ (2004). Acute respiratory failure from abused substances. J. Intensive Care. Med..

[32] Reutershan J, Ley K (2004). Bench-to-bedside review: acute respiratory distress syndrome – how neutrophils migrate into the lung. Crit. Care..

[33] Wang L (2016). Anticancer properties of nimbolide and pharmacokinetic considerations to accelerate its development. Oncotarget.

[34] Glinsukon T, Somjaree R, Piyachaturawat P, Thebtaranonth Y (1986). Acute toxicity of nimbolide and nimbic acid in mice, rats and hamsters. Toxicol. Lett..

[35] Kerksick C, Willoughby D (2005). The Antioxidant role of glutathione and N-acetyl-cysteine supplements and exercise-induced oxidative stress. J. Int. Soc. Sports Nutr..

[36] Anatoliotakis N (2013). Myeloperoxidase: expressing inflammation and oxidative stress in cardiovascular disease. Curr. Top. Med. Chem..

[37] West AP (2011). TLR signaling augments macrophage bactericidal activity through mitochondrial ROS. Nature.

[38] Hanafy KA, Krumenacker JS, Murad F (2001). NO, nitrotyrosine, and cyclic GMP in signal transduction. Med. Sci. Monit..

[39] Korhonen R, Lahti A, Kankaanranta H, Moilanen E (2005). Nitric oxide production and signaling in inflammation. Curr. Drug. Targets Inflamm. Allergy.

[40] Grommes J, Soehnlein O (2011). Contribution of neutrophils to acute lung injury. Mol. Med..

[41] Kato A, Hulse KE, Tan BK, Schleimer RP (2013). B-lymphocyte lineage cells and the respiratory system. J. Allergy Clin. Immunol..

[42] Moldoveanu B (2009). Inflammatory mechanisms in the lung. J. Inflamm. Res..

[43] Nakagome K, Nagata M (2011). Pathogenesis of airway inflammation in bronchial asthma. Auris Nasus Larynx.

[44] Bryant AH, Spencer-Harty S, Owens SE, Jones RH, Thornton CA (2017). Interleukin 4 and interleukin 13 downregulate the lipopolysaccharide-mediated inflammatory response by human gestation-associated tissues. Biol. Reprod..

[45] Lundblad LKA (2005). Tumor necrosis factor–α overexpression in lung disease. Am. J. Respir. Crit. Care. Med..

[46] Parameswaran N, Patial S (2010). Tumor necrosis factor-α signaling in macrophages. Crit. Rev. Eukaryot. Gene Expr..

[47] Liu Z (2013). Inhibitory effects of rosiglitazone on paraquat-induced acute lung injury in rats. Acta Pharmacol. Sin..

[48] Shi L, Kishore R, McMullen MR, Nagy LE (2002). Lipopolysaccharide stimulation of ERK1/2 increases TNF-alpha production via Egr-1. Am. J. Physiol. Cell. Physiol..

[49] Zidi I, Mestiri S, Bartegi A, Amor NB (2010). TNF-alpha and its inhibitors in cancer. Med. Oncol..

[50] Li Y (2016). Angiotensin-converting enzyme 2 prevents lipopolysaccharide-induced rat acute lung injury *via* suppressing the ERK1/2 and NF-κB signaling pathways. Sci. Rep..

[51] Gao Z, He Q, Peng B, Chiao PJ, Ye J (2006). Regulation of nuclear translocation of HDAC3 by IkappaBalpha is required for tumor necrosis factor inhibition of peroxisome proliferator-activated receptor gamma function. J. Biol. Chem..

[52] Zhu H, Shan L, Schiller PW, Mai A, Peng T (2010). Histone deacetylase-3 activation promotes tumor necrosis factor-α (TNF-α) expression in cardiomyocytes during lipopolysaccharide Stimulation. J. Biol. Chem..

