# Supplementary material for: Nimbolide protects against endotoxin-induced acute respiratory distress syndrome by inhibiting TNF-α mediated NF-κB and HDAC-3 nuclear translocation
Source: Cell Death Dis. 2019 Jan 28;10(2):81. doi: 10.1038/s41419-018-1247-9 (PMC6349848; doi:10.1038/s41419-018-1247-9)
Supplement: Supplementary file 1 — Supplementary data [file 41419_2018_1247_MOESM1_ESM.docx]

**Nimbolide protects against endotoxin-induced acute respiratory distress syndrome by inhibiting TNF-α mediated NF-κB and HDAC-3 nuclear translocation**

Venkatesh Pooladanda^1^, Sowjanya Thatikonda^1^, Swarna Bale^1^, Bijay Pattnaik^2^, Dilep Kumar Sigalapalli^3^, Nagendra Babu Bathini^3^, Shashi Bala Singh^1^ and Chandraiah Godugu^1*^

^1^Department of Regulatory Toxicology, National Institute of Pharmaceutical Education and Research (NIPER), Balanagar, Hyderabad, Telangana-500037, India.

^2^Centre of Excellence in Asthma & Lung Disease and Molecular Immunogenetics Laboratory, CSIR‐Institute of Genomics and Integrative Biology-110007, New Delhi, India.

^3^Department of Medicinal Chemistry, National Institute of Pharmaceutical Education and Research (NIPER), Balanagar, Hyderabad, Telangana-500037, India.

**Running title:** Nimbolide ameliorates LPS induced acute respiratory distress syndrome

^*^Correspondence:

Dr. Chandraiah Godugu, Assistant Professor,

Department of Regulatory Toxicology,

National Institute of Pharmaceutical Education and Research (NIPER),

Balanagar, Hyderabad,

Telangana, India-500037

Telephone: 040-23073741 Fax: 040-23073751

E-mail: [chandragodugu@gmail.com](mailto:chandragodugu@gmail.com),

[chandra.niperhyd@gov.in](mailto:chandra.niperhyd@gov.in)

**SUPPLEMENTARY METHODS**

***In silico* molecular modeling**

1. ***Computational modeling and protein docking***

The crystal structures of the human TNF-α (PDB ID: 2AZ5)^1^ was used for molecular docking studies. For the ligand docking, the standard precision mode was selected. Docking was performed by using the standard protocol implemented in Maestro, version 9.7 and the ligands were docked against the active sites of targeted proteins. No constraints were defined for the docking runs.

1. ***Computational methods***

***Protein Preparation and Grid Generation***

The coordinates for the TNF-α were downloaded from the RCSB protein data bank. The protein structures were processed with the Protein Preparation Wizard in the Schrödinger suite. The protein structure integrity was checked and adjusted. The missing residues and loop segments near the active site were added using Prime module of Schrödinger suite. The receptor was prepared for docking by the addition of hydrogen atoms and the removal of cocrystallized molecule. Active site water molecules outside 5.0 Å from the ligand were removed. The bound ligand was used to specify the active site in the TNF-α protein. Visualization and characterization of the catalytic binding site were done by SiteMap module of Maestro 9.7. The receptor grid for target protein was prepared with the help of OPLS_2005 force field. The grid center was set to be the centroid of the active site and the cubic grid had a size of 20 Å.

***Ligand Preparation***

The ligand molecules were built on Maestro Molecule Builder of Schrödinger. The built molecules were optimized using OPLS_2005 force field in the LigPrep module of Schrödinger software. All possible protomers and ionization states were enumerated for ligands using Ionizer at a pH of 7.4. Tautomeric states were generated for chemical groups with possible prototropic tautomerism.

***Molecular Docking***

Molecular docking studies were performed by using a GLIDE docking module of Schrödinger suite. The prepared ligands were docked into the generated receptor grids using Glide SP docking precision. Each complex was analyzed for interactions and the 3D poses were taken. 3D poses demonstrates the molecular recognition interactions. All the 3D figures were obtained using Schrödinger Suite 2014-1 and PyMOL v0.99.

***Prime MM/GBSA binding energy calculations***

The MM/GBSA (Molecular mechanics/generalized born surface area) analysis was used to calculate ligand-binding energies based on docking complex, using the MM/GBSA technology available in Prime module of Schrödinger software. The protein ligand complexes obtained from molecular docking were subjected to MM/GBSA calculations. The relative binding free energy **Δ***G*_bind_ was estimated according to following equation:

**Δ***G*_bind =_ *E*_complex_ (minimized) - [*E*_ligand_ (unbound, minimized) +

*E*_receptor_ (unbound, minimized)]

Where, **Δ***G*_bind_ is the calculated relative free energy which includes both ligand and receptor strain energy. E_complex_ (minimized) is the MM/GBSA energy of the minimized complex, and E_ligand_ (unbound, minimized) is the MM/GBSA energy of the ligand after removing it from the complex and allowing it to relax. E_receptor_ (unbound, minimized) is the MM/GBSA energy of protein after separating it from the ligand.

**Real-Time PCR**

**Table S1.** Primers sequence

| **Gene** | **Primer** | **Sequence** |
| --- | --- | --- |
| NF-κB1 | Forward primer | GACCACTGCTCAGGTCCACT |
|  | Reverse primer | CATCTATGTGCTGCCTCGT |
| HDAC-3 | Forward primer | CAATCTCAGCATTCGAGGACATG |
|  | Reverse primer | GCAACATTTCGGACAGTGTAGCC |
| TNF-α | Forward primer | GACGTGGAAGTGGCAGAAGAG |
|  | Reverse primer | TGCCACAAGCAGGAATGAGA |
| 18S rRNA | Forward primer | GTAACCCGTTGAACCCCATT |
|  | Reverse primer | CCATCCAATCGGTAGTAGCG |

**SUPPLEMENTARY RESULTS**

***In silico* physicochemical and ADME/T studies**

Physicochemical and ADME/T properties were calculated using Qikprop 4.6 module of Schrödinger suite 2015-4. It predicts both physicochemical significant descriptors and pharmacokinetical important properties of the molecules. QikProp enumerates the comparative ranges of molecules properties with those of known drugs. Schrödinger software was used to predict the nimbolide phyicochemical and ADME/T profile and the results were represented in **Table S2**.

Nimbolide showed significant values for the physicochemical properties analyzed and exhibited druglike characteristics. It has appropriate logP value for biological efficacy and no violation in the recommended ranges of physicochemical and ADME/T parameters. The other associated factor, blood brain permeability and percent human oral absorption also in the acceptable range for this compound. All these *in silico* parameters evaluated for nimbolide were within the acceptable range defined for human use. Thus, from these studies we observed that the nimbolide has good drug likeliness properties.

**Table S2.** Physicochemical properties and **ADME/T** Profile of nimbolide.

| **Compound name** | **Parameters** | | | | | | | | | | |
| --- | --- | --- | --- | --- | --- | --- | --- | --- | --- | --- | --- |
|  |  | | | | | | | | | | |
|  | **Rule of five** | **PSA**  **(1)** | **QPlogKhsa**  **(2)** | **Predicted octanol/water partition coefficient**  **(3)** | **Predicted polarizability**  **(4)** | **Predicted**  **CNS**  **activity**  **(5)** | **Predicted apparent Caco-2 cell permeability in nm/sec**  **(6)** | **Predicted brain/blood partition coefficient**  **(7)** | **Predicted skin permeability**  **(8)** | **Ionization potential**  **(9)** | **Percent human oral absorption**  **(10)** |
| **Nimbolide** | No violation | 109.37 | -0.03 | 3.03 | 48.00 | -1 | 940.65 | -0.51 | -2.67 | 9.34 | 100.00 |

The recommended ranges for some of the computed Physicochemical and **ADME/T** parameters showed in **Table S2** were mentioned below.

1. Van der Waals surface area of polar nitrogen and oxygen atoms and carbonyl carbon atoms (**PSA**): 7.0 to 200.0
2. Prediction of binding to human serum albumin (**QPlogKhsa**): –1.5 to 1.5
3. Predicted octanol/water partition coefficient (**QPlogPo/w**): –2.0 to 6.5
4. Predicted polarizability in cubic angstroms (**QPpolrz**): 13.0 to 70.0
5. Predicted central nervous system activity (**CNS**): –2 (inactive) to +2 (active)
6. Predicted apparent Caco-2 cell permeability (**QPPCaco**) in nm/sec: <25 is poor, whereas >500 is great
7. Predicted brain/blood partition coefficient (**QPlogBB**):–3.0 to 1.2
8. Predicted skin permeability (**QPlogKp**): –8.0 to –1.0
9. PM3 calculated ionization potential (**IP(ev)†**): 7.9 to 10.5
10. Predicted human oral absorption on 0 to 100% scale: >80% is high, whereas <25% is poor

**SUPPLEMENTAL FIGURES AND TABLES**


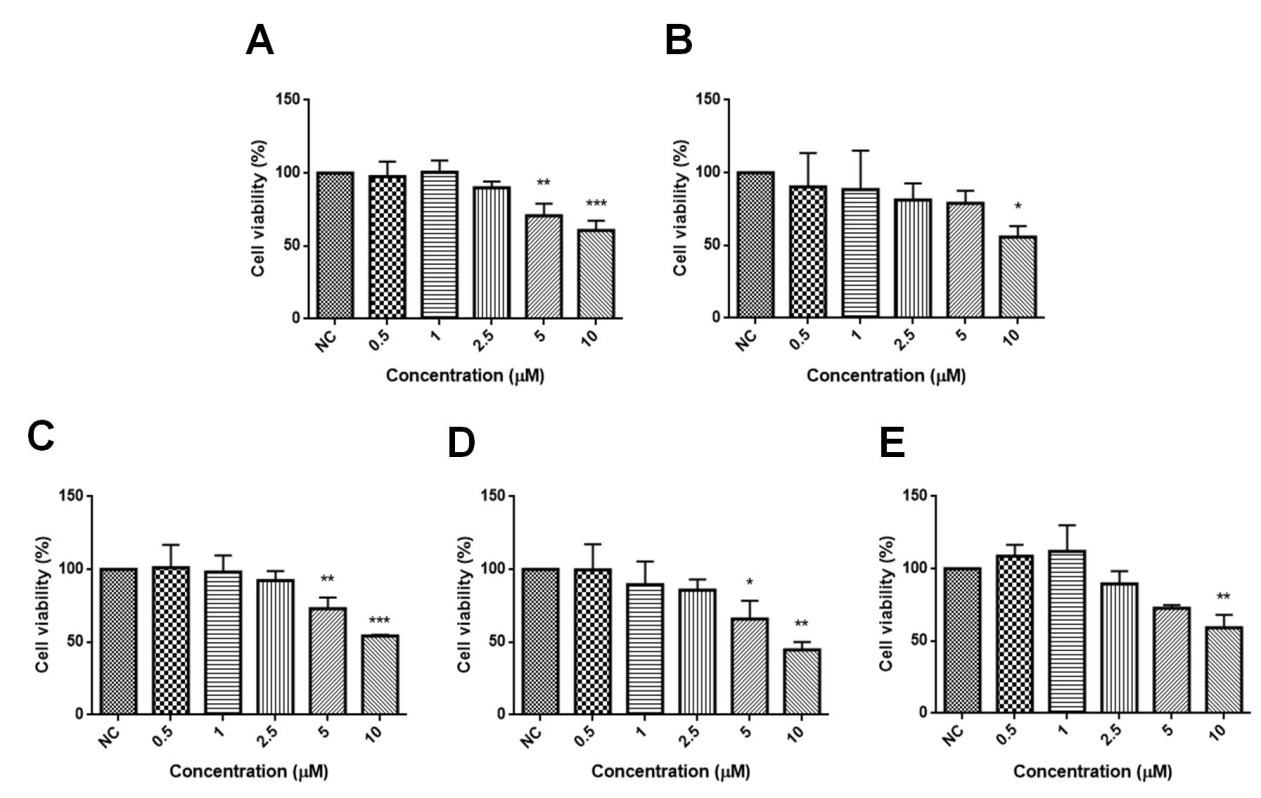


**Figure S1. Effect of nimbolide on cell viability of macrophages and lung epithelial cells.** (**A**) RAW 264.7, (**B**) THP-1, (**C**) MLE-12, (**C**) A549, and (**D**) BEAS-2B cells were seeded in 96-well plate. Then cells were treated with various concentrations of nimbolide (0.5-10 µM) and incubated for 24 h. Percentage cell viability was measured by MTT assay. Data represented as mean ± SEM (n=3 independent experiments). **P*<0.05, ***P*<0.01 and ****P*<0.001 are significantly different from the normal control (NC) group.

**
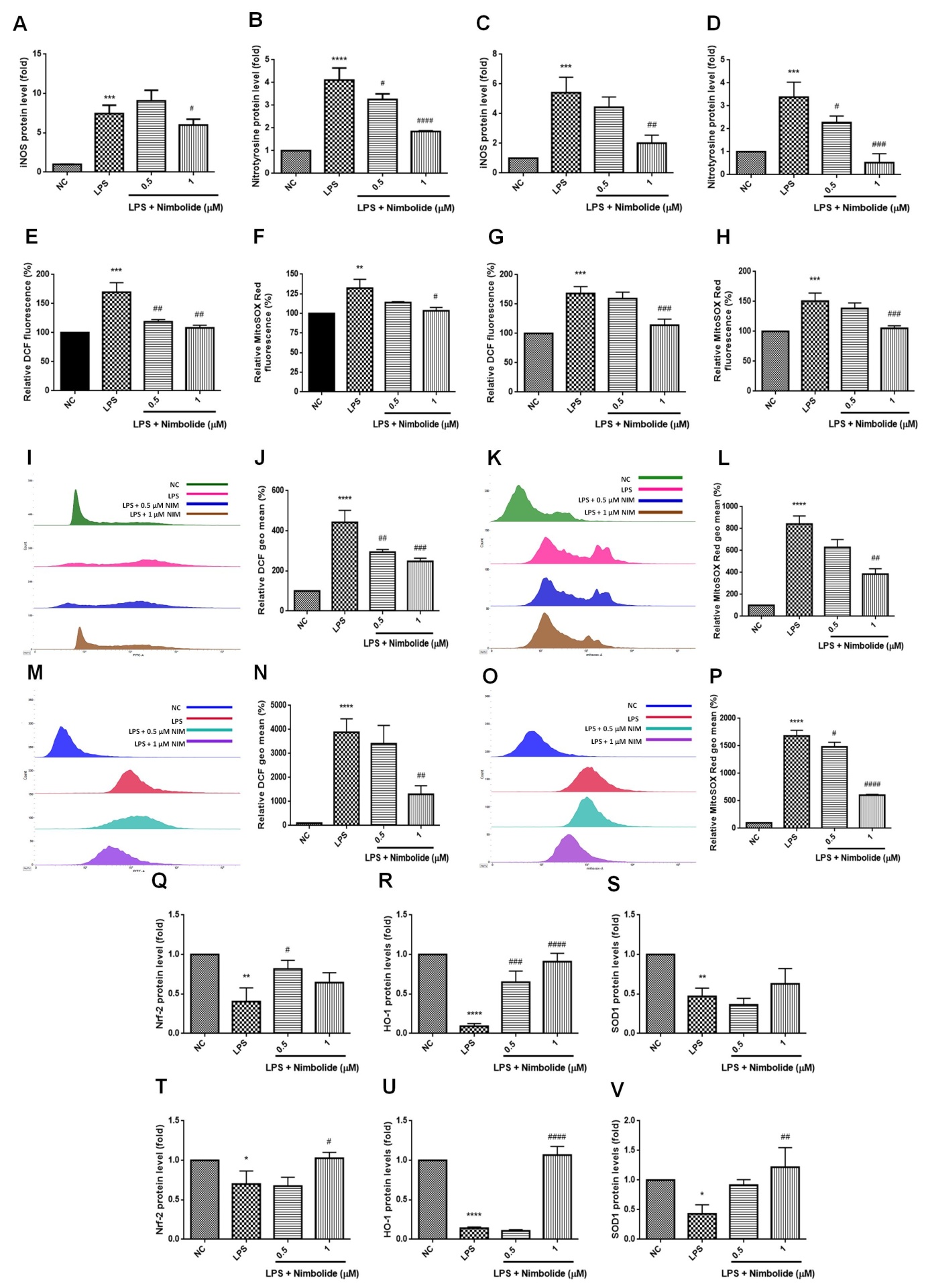
**

**Figure 2S. Nimbolide ameliorates nitrosative-oxidative stress.** Both (**A-B**) RAW 264.7 and (**C-D**) differentiated THP-1 cells were pre-treated with nimbolide for 24 h and stimulated with LPS (1 µg/ml) for 24 h. Whole cell protein was isolated from both cell lines and western blot was performed. The densitometric analysis was performed by ImageJ, NIH, USA software. The protein levels of iNOS and nitrotyrosine were represented as the fold over NC. Intracellular and mitochondrial ROS (mROS) levels were determined by DCFDA and MitoSOX Red staining, respectively. Both the cell lines were pre-treated with nimbolide for 24 h and stimulated with LPS (1 µg/ml) for 30 min. The relative fluorescent intensity of DCF and MitoSOX Red was measured by multimode plate reader in both (**E-F**) RAW 264.7 and (**G-H**) differentiated THP-1 cells, respectively. Additionally, (**I-L**) RAW 264.7 and (**M-P**) differentiated THP-1 cells subjected to flowcytometric analysis to determine the relative geo mean of DCF and MitoSOX Red. Cells were pre-treated with nimbolide for 24 h and stimulated with LPS (1 µg/ml) for 24 h, followed by whole cell lysate extraction to determine the protein expression (the fold over NC) of Nrf-2, HO-1, and SOD-1 in both (**Q-S**) RAW 264.7 and (**T-V**) differentiated THP-1 cells. Data represented as mean ± SEM (n=3 independent experiments). **P*<0.05, ***P*<0.01, ****P*<0.001, and *****P*<0.0001 are significantly different from the NC group; ^#^*P*<0.05, ^##^*P*<0.01, ^###^*P*<0.001, and ^####^*P*<0.0001 are significantly different from the LPS group.

**
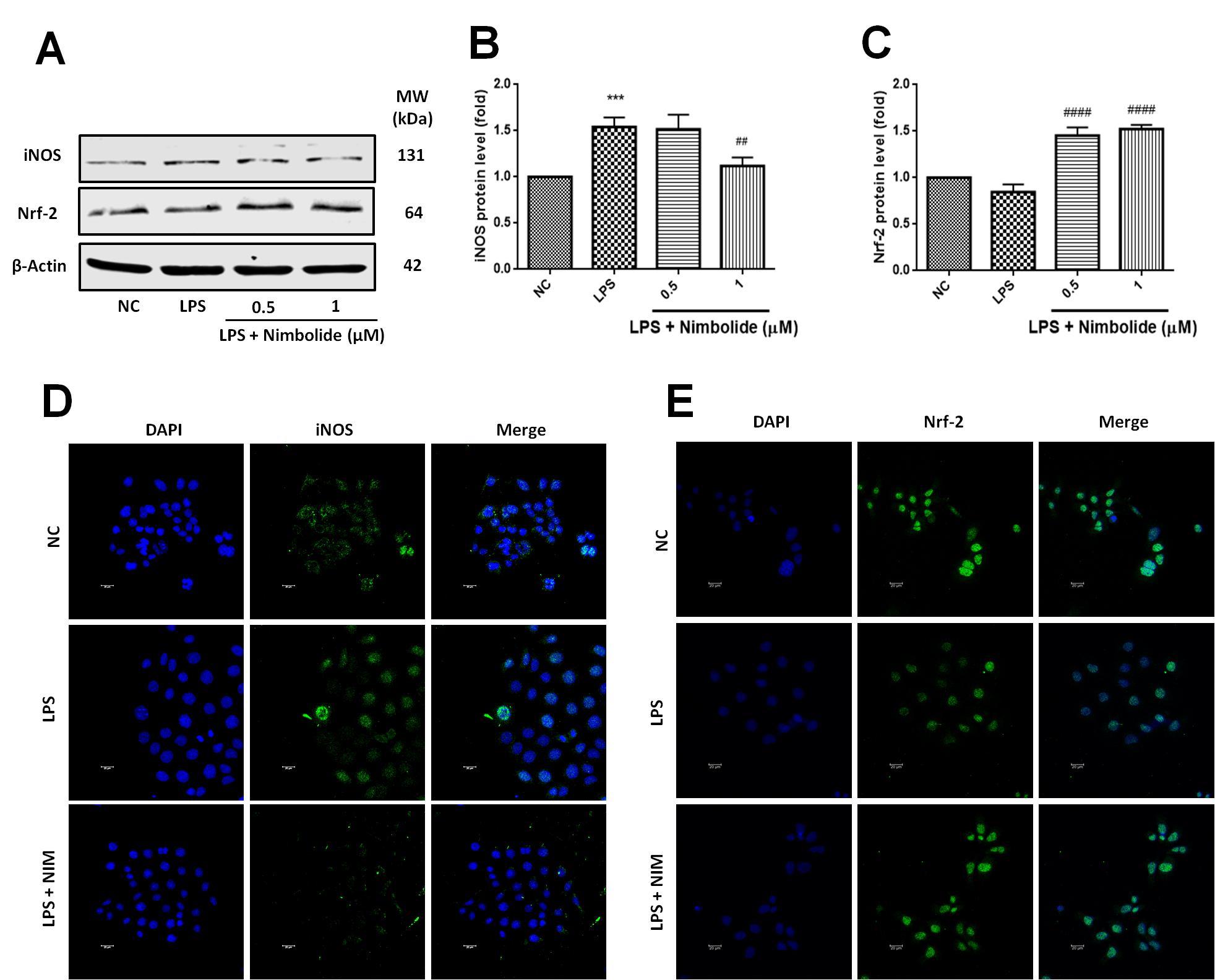
**

**Figure 3S. Nimbolide inhibits LPS induced nitrosative and oxidative stress in mouse lung epithelial cells.** MLE-12 cells were pre-treated with nimbolide (0.5 and 1 μM) and stimulated with LPS (1 μg/ml) for 24 h. (**A-C**) Whole cell protein was extracted and western blot analysis was performed. The iNOS and Nrf-2 expressions were determined and quantified by densitometric analysis using ImageJ software and data expressed as the fold over NC. Additionally, confocal analysis was performed to determine the expression of iNOS and Nrf-2 in MLE-12 cells. The images were captured at ×400 magnification. Data represented as mean ± SEM (n=3 independent experiments). ****P*<0.001 is significantly different from the NC group; ^##^*P*<0.01 and ^####^*P*<0.0001 are significantly different from the LPS group.


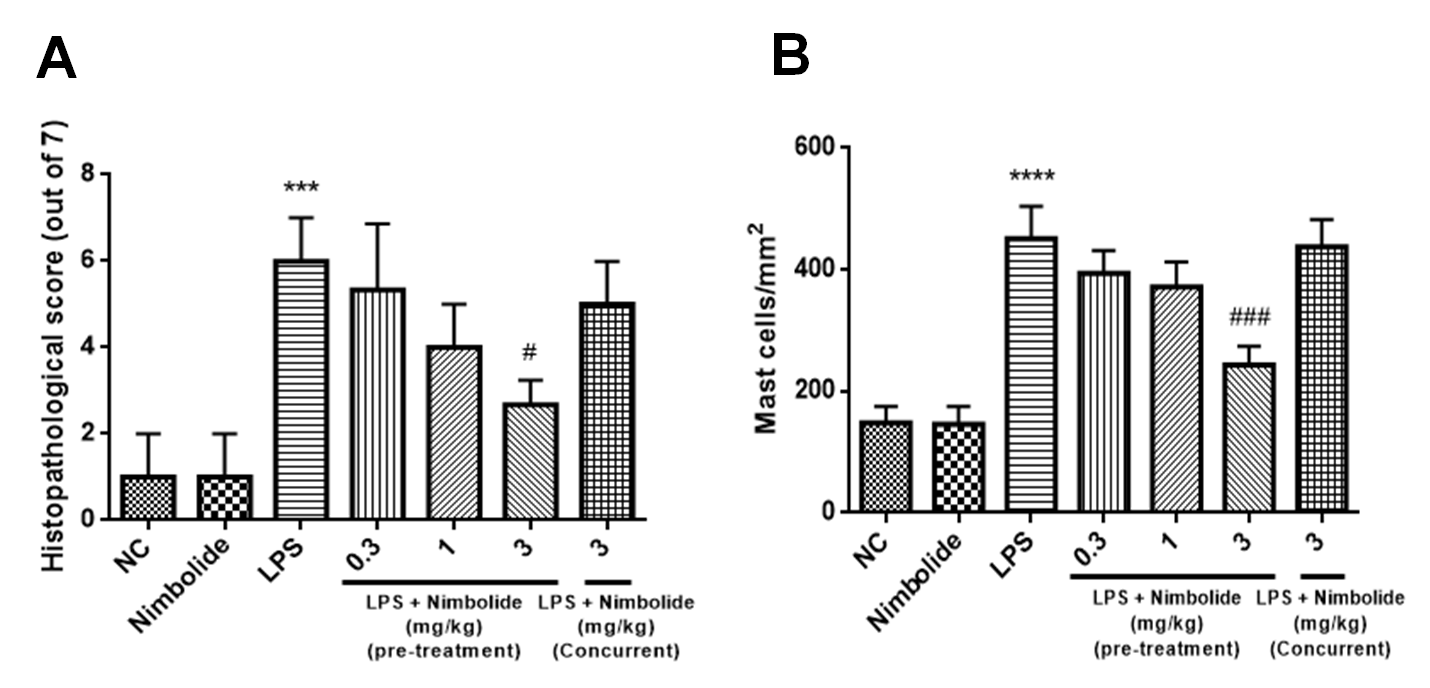


**Figure S4. Nimbolide ameliorates LPS induced alveolar changes by reducing neutrophil infiltration and mast cell density.** Lungs were collected and sectioned followed by H & E staining and TB staining. (**A**) Total histopathological scores were determined for all groups after H&E staining. (**B**) Mast cells were quantified from TB staining slides. Data represented as mean ± SEM (n=8 animals per group). ****P*<0.001 and *****P*<0.0001 are significantly different from the NC group; ^#^*P*<0.05 and ^###^*P*<0.001 are significantly different from the LPS group.


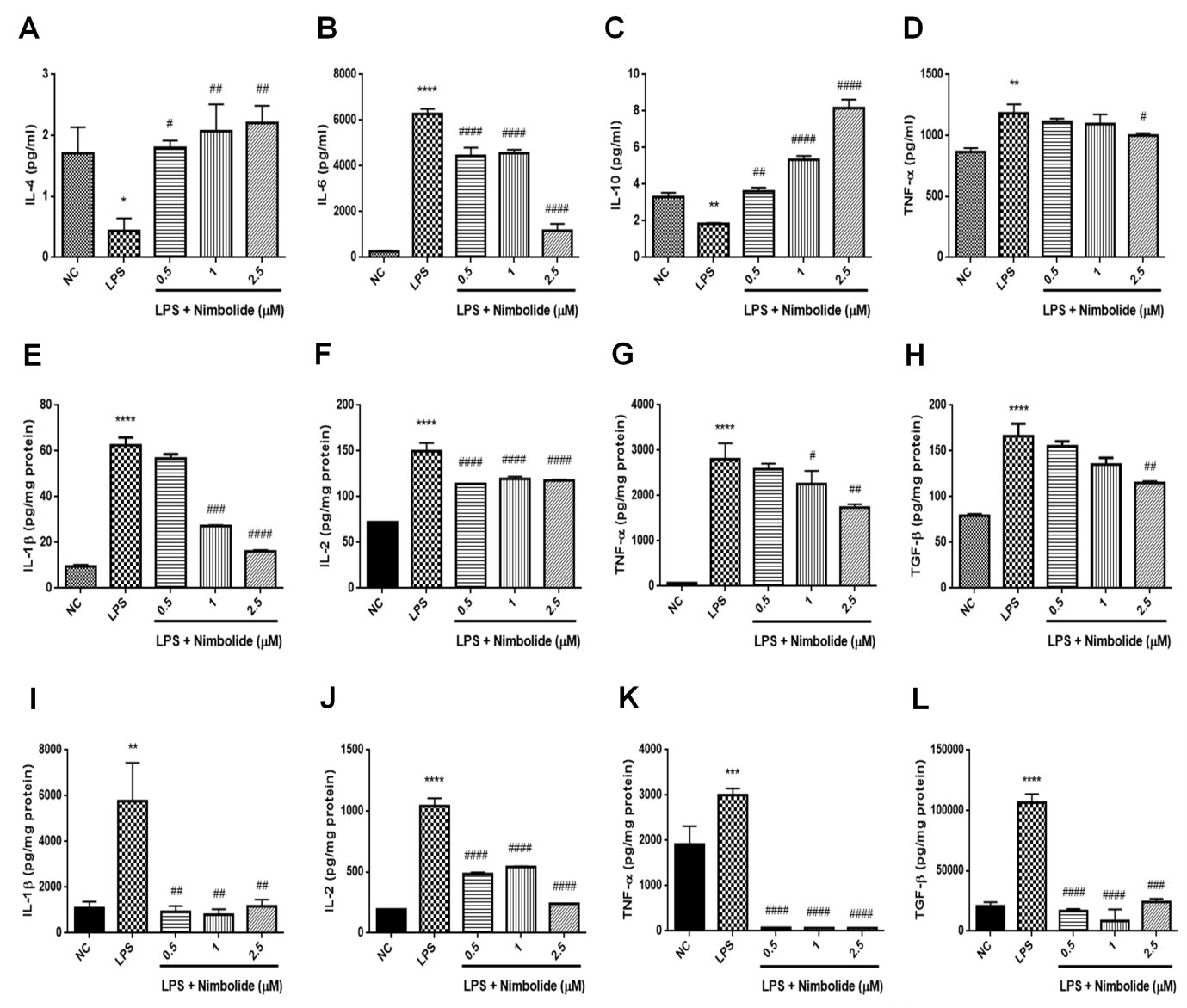


**Figure S5. Nimbolide modulates the pro- and anti-inflammatory cytokines.** Cells were pre-treated with nimbolide for 24 h and stimulated with LPS (1 µg/ml) for 12 h. (**A-D**) Cell lysate from RAW 264.7 cells was prepared to measure the cytokines and chemokines. The pro-inflammatory cytokines (IL-6 and TNF-α) and anti-inflammatory cytokines (IL-4 and IL-10) expression were determined by multiplex. Whereas, (**E-H**) RAW 264.7 and (**I-L**) A549 cell culture supernatants were subjected to ELISA to determine IL-1β, IL-2, TNF-α, and TGF-β cytokines expression. Data represented as mean ± SEM (n=3 independent experiments). **P*<0.05, ***P*<0.01, ****P*<0.001, and *****P*<0.0001 are significantly different from the NC group; ^#^*P*<0.05, ^##^*P*<0.01, ^###^*P*<0.001, and ^####^*P*<0.0001 are significantly different from the LPS group.


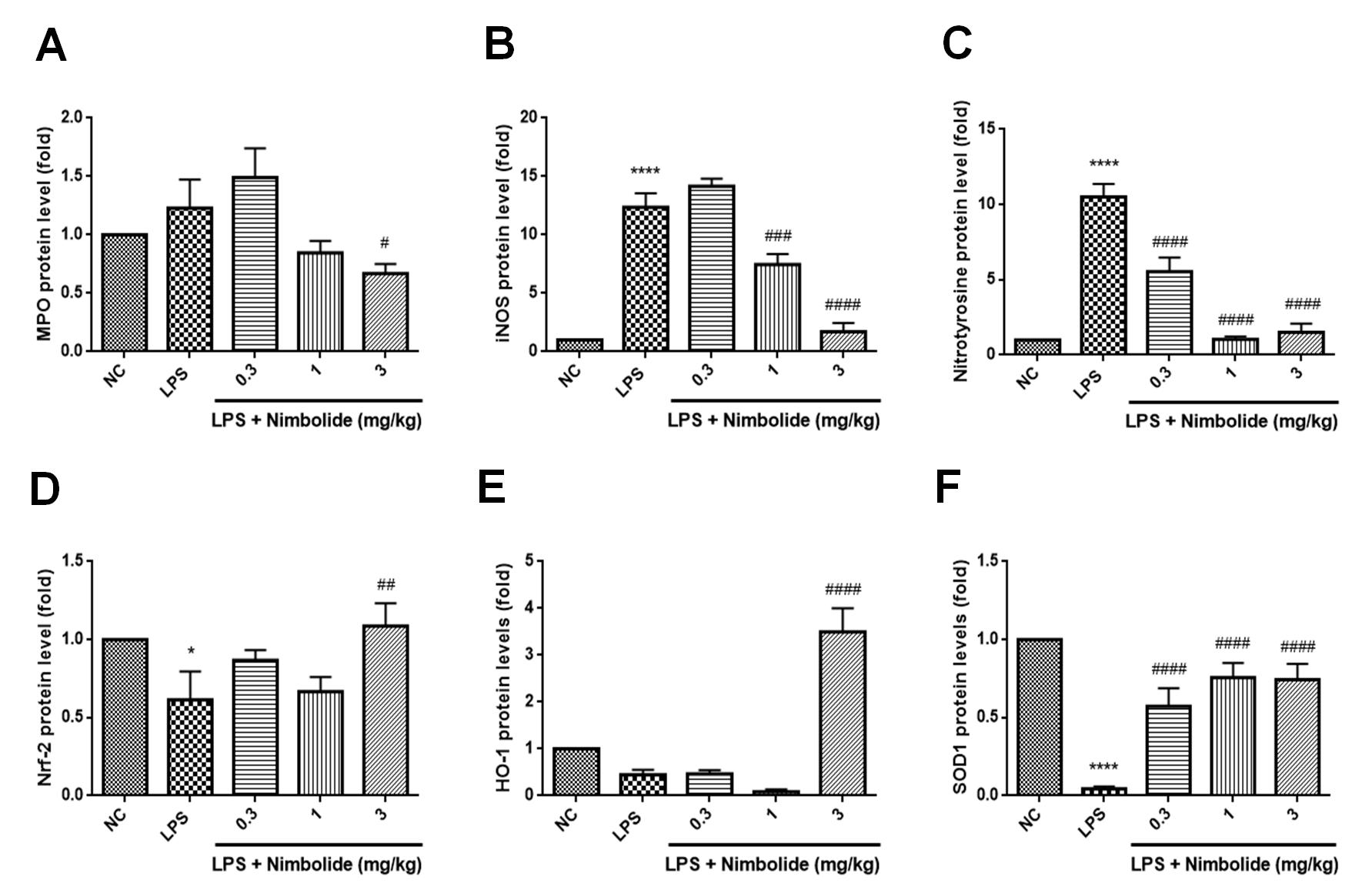


**Figure S6. Nimbolide exhibits anti-oxidant effect in lung tissues against LPS induced inflammation.** Mice were pre-treated with nimbolide (0.3, 1 and 3 mg/kg) for 5 days. Later, inflammation was induced by LPS (50 µg) administrated through oropharyngeal instillation. Protein was isolated from lung tissues and western blotting was performed. Densitometric analysis was performed to determine the protein levels of (**A**) MPO, (**B**) iNOS, (**C**) nitrotyrosine, (**D**) Nrf-2, (**E**) HO-1, and (**F**) SOD-1. The bar charts are the quantitative analysis of the corresponding immunoblots and the data expressed as the fold over NC. Data presented as mean ± SEM (n=8 animals per group). **P*<0.05 and *****P*<0.0001 are significantly different from the NC group; ^#^*P*<0.05, ^##^*P*<0.01, ^###^*P*<0.001, and ^####^*P*<0.0001 are significantly different from the LPS group.

**
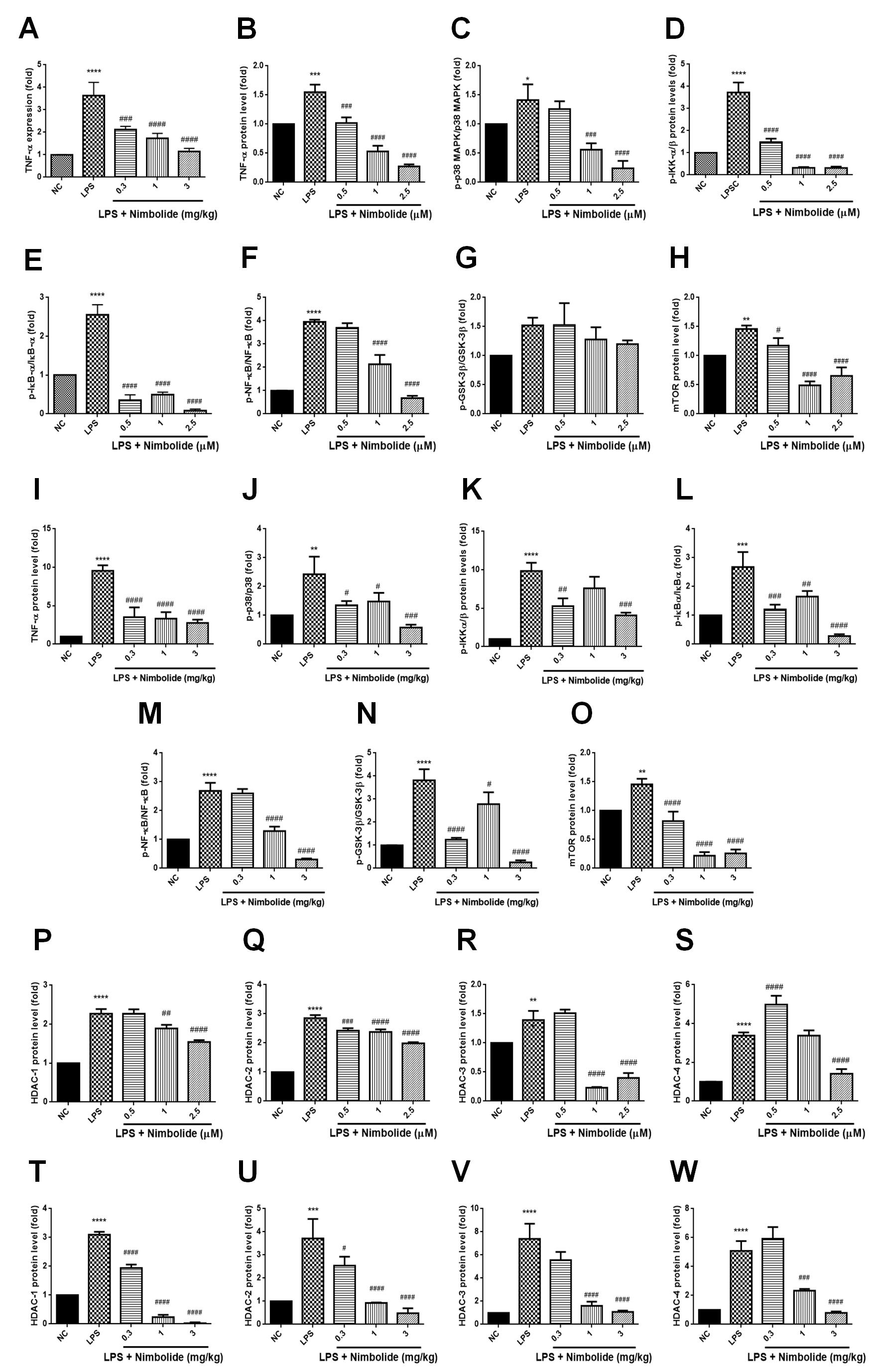
**

**Figure S7. Nimbolide shows anti-inflammatory effect by ameliorating LPS induced ARDS. (A**) 5 µm sections were subjected to IHC and determined the expression of TNF-α in lung tissues. The quantitative analysis was performed by ImageJ Fiji. A549 cells were pre-treated with nimbolide for 24 h and stimulated with LPS (1 µg/ml) for 12 h. Whole cell protein was isolated and immunoblotting was performed and protein levels were quantified by densitometric analysis. The protein levels of (**B**) TNF-α, (**C**) p-p38 MAPK, (**D**) p-IKK-α/β, (**E**) p-IκB, (**F**) p-NF-κB, (**G**) p-GSK-3β, and (**H**) mTOR were expressed as the fold over NC. After 12 h of LPS post-exposure, protein was isolated from lungs and western blot was performed. The protein levels of (**I**) TNF-α, (**J**) p-p38 MAPK, (**K**) p-IKK-α/β, (**L**) p-IκB, (**M**) p-NF-κB, (**N**) p-GSK-3β, and (**O**) mTOR were expressed as the fold. Nuclear protein was isolated from both (**P-S**) A549 cells and (**T-W**) lung tissues, followed by western blotting, protein levels of HDAC-1, 2, 3, and 4 represented as the fold change over NC. The bar charts are the quantitative analysis of the corresponding immunoblots, and the data expressed as the fold over NC. Data represented as mean ± SEM (n=3 independent experiments, *in vitro*; n=8 animals per group, *in vivo*). **P*<0.05, ***P*<0.01, ****P*<0.001, and *****P*<0.0001 are significantly different from the NC group; ^#^*P*<0.05, ^##^*P*<0.01, ^###^*P*<0.001, and ^####^*P*<0.0001 are significantly different from the LPS group.


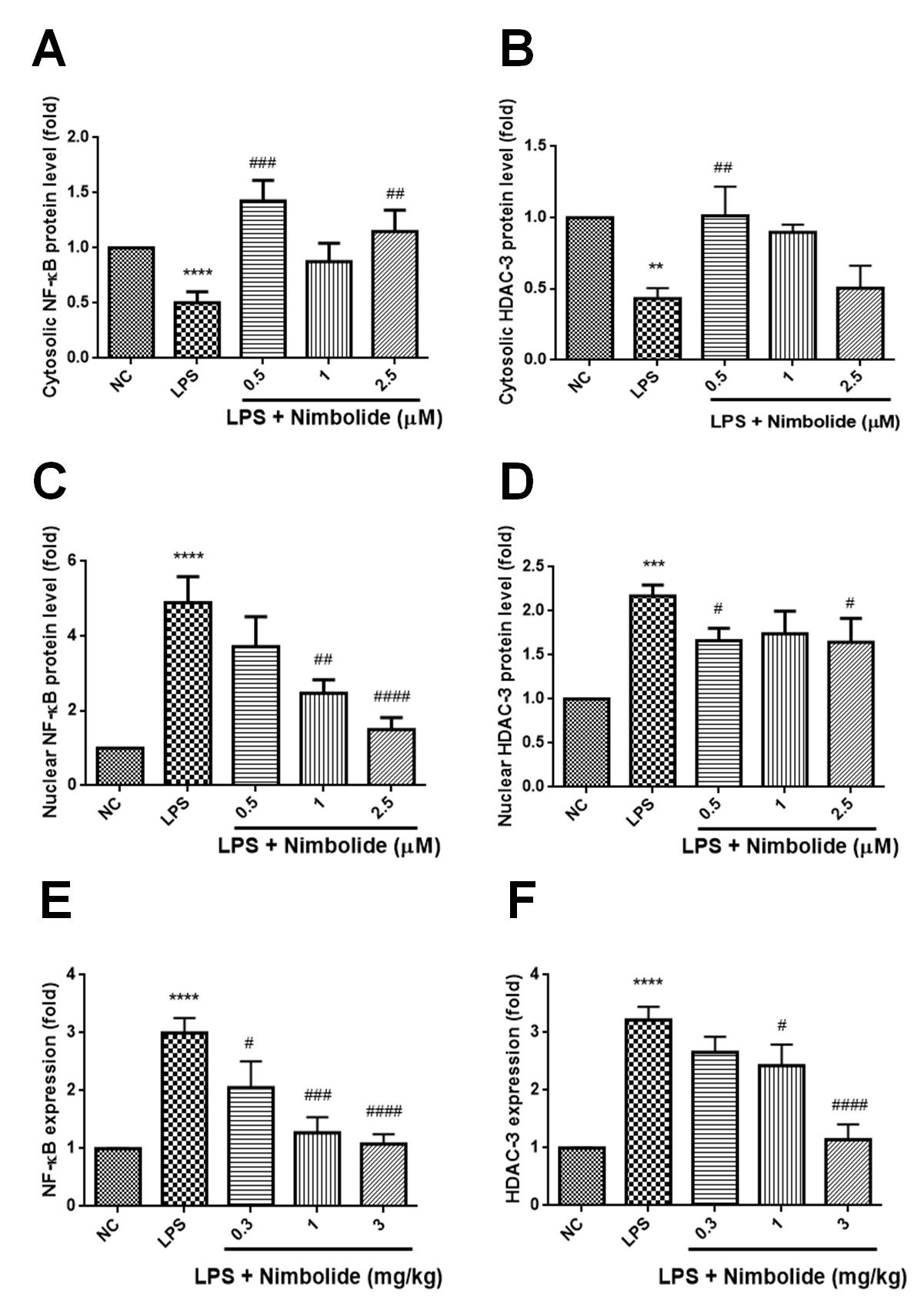


**Figure S8. Nimbolide inhibits nuclear translocation of NF-κB and HDAC-3.** A549 cells were pre-treated with nimbolide for 24 h and stimulated with LPS (1 µg/ml) for 30 min. Both cytosolic and nuclear proteins were extracted and immunoblot analysis was performed. Densitometric analysis was used to quantify the (**A-B**) cytosolic and (**C-D**) nuclear protein levels of NF-κB and HDAC-3 as the fold change over NC. Mouse lung tissue sections were subjected to IHC and determined the expression of (**E**) NF-κB and (**F**) HDAC-3. The quantitative analysis was performed by ImageJ Fiji software and results were expressed as the fold change over NC. Data represent as mean ± SEM (n=3 independent experiments, *in vitro*; n=8 animals per group, *in vivo*). ^**^*P*<0.01 and ^****^*P*<0.0001 are significantly different from the NC group; ^#^*P*<0.05, ^##^*P*<0.01, ^###^*P*<0.001, and ^####^*P*<0.0001 are significantly different from the LPS group.


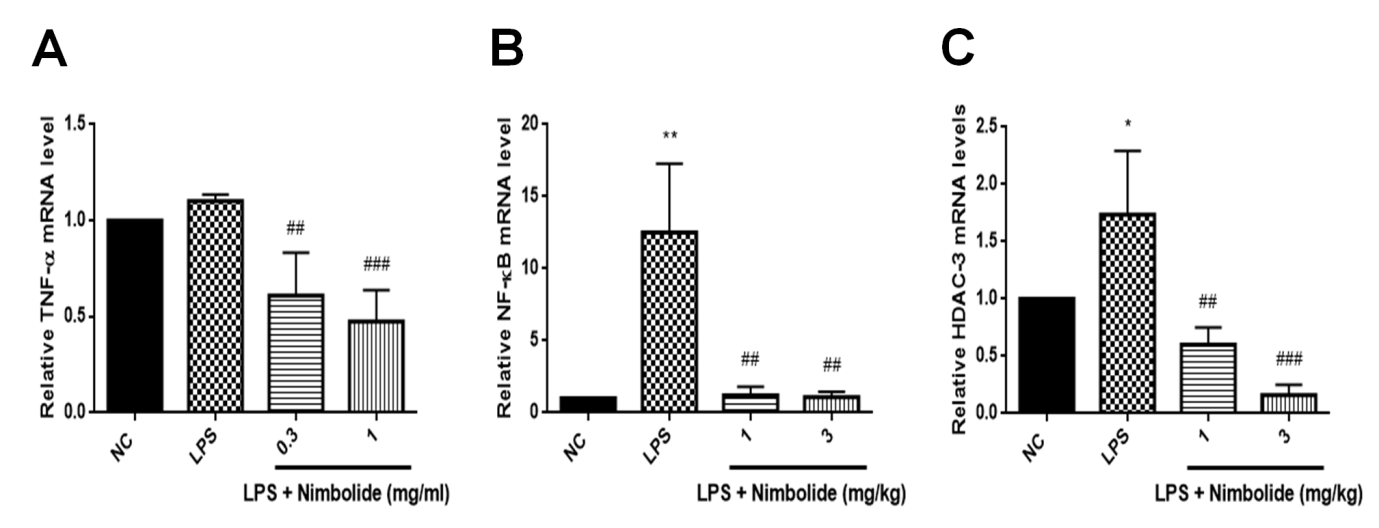


**Figure S9. Nimbolide represses the LPS induced inflammation regulated genes transcription.** The RNA was isolated from lung tissues and relative mRNA levels of **(A)** TNF-α, **(B)** NF-κB and **(C)** HDAC-3 were determined by RT-PCR. Data represent as mean ± SEM (n=8 animals per group). ^*^*P*<0.05 and ^**^*P*<0.01 are significantly different from the NC group; ^##^*P*<0.01 and ^###^*P*<0.001 are significantly different from the LPS group.

**
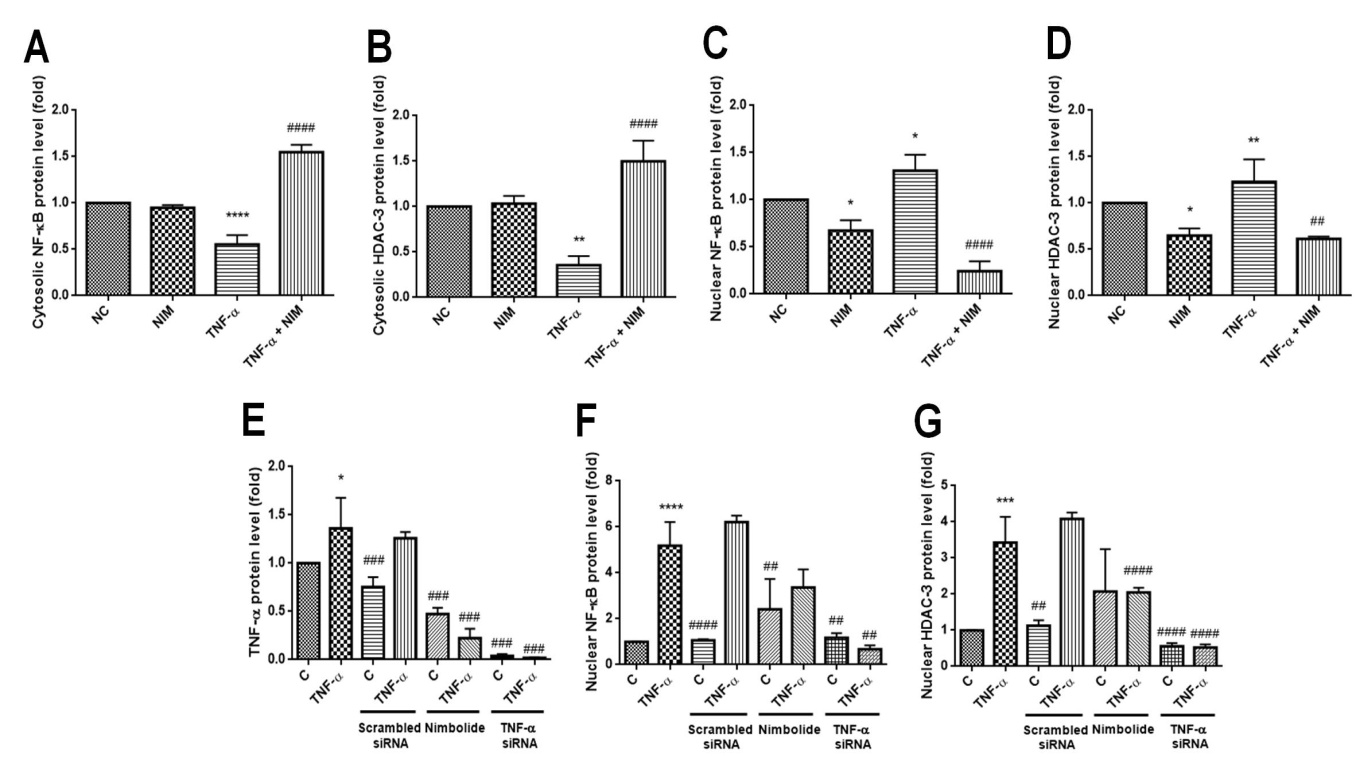
**

**Figure S10. Nimbolide inhibits TNF-α induced nuclear translocation of NF-κB and HDAC-3.** A549 cells were pre-treated with nimbolide for 24 h and stimulated with TNF-α (10 ng/ml) for 30 min. Here, NIM (nimbolide alone) group (2.5 µM) didn’t receive TNF-α. The protein was isolated from cells and immunoblotting was performed. The blots were quantified by densitometric analysis using ImageJ software and protein levels of (**A-B**) cytosolic and (**C-D**) nuclear NF-κB and HDAC-3 were represented as the fold change over NC. TNF-α protein expression was silenced by TNF-α siRNA and scrambled siRNA used as a control. BEAS-2B cells were transfected with TNF-α and scrambled siRNA (50 nM) for 24 h. In another set of group, cells were pre-treated with nimbolide (2.5 μM) for 24 h. Then cells were stimulated with TNF-α (10 ng/ml) for 30 min. The expression of TNF-α, NF-κB and HDAC-3 levels were measured by western blotting. All the blots were quantified by densitometric analysis and represented as the fold change over NC. Data represent as mean ± SEM (n=3 independent experiments). ^*^*P*<0.05, ^**^*P*<0.01, ^***^*P*<0.001, and ^****^*P*<0.0001 are significantly different from the NC group; ^##^*P*<0.01, ^###^*P*<0.001, and ^####^*P*<0.001 are significantly different from the TNF-α group.


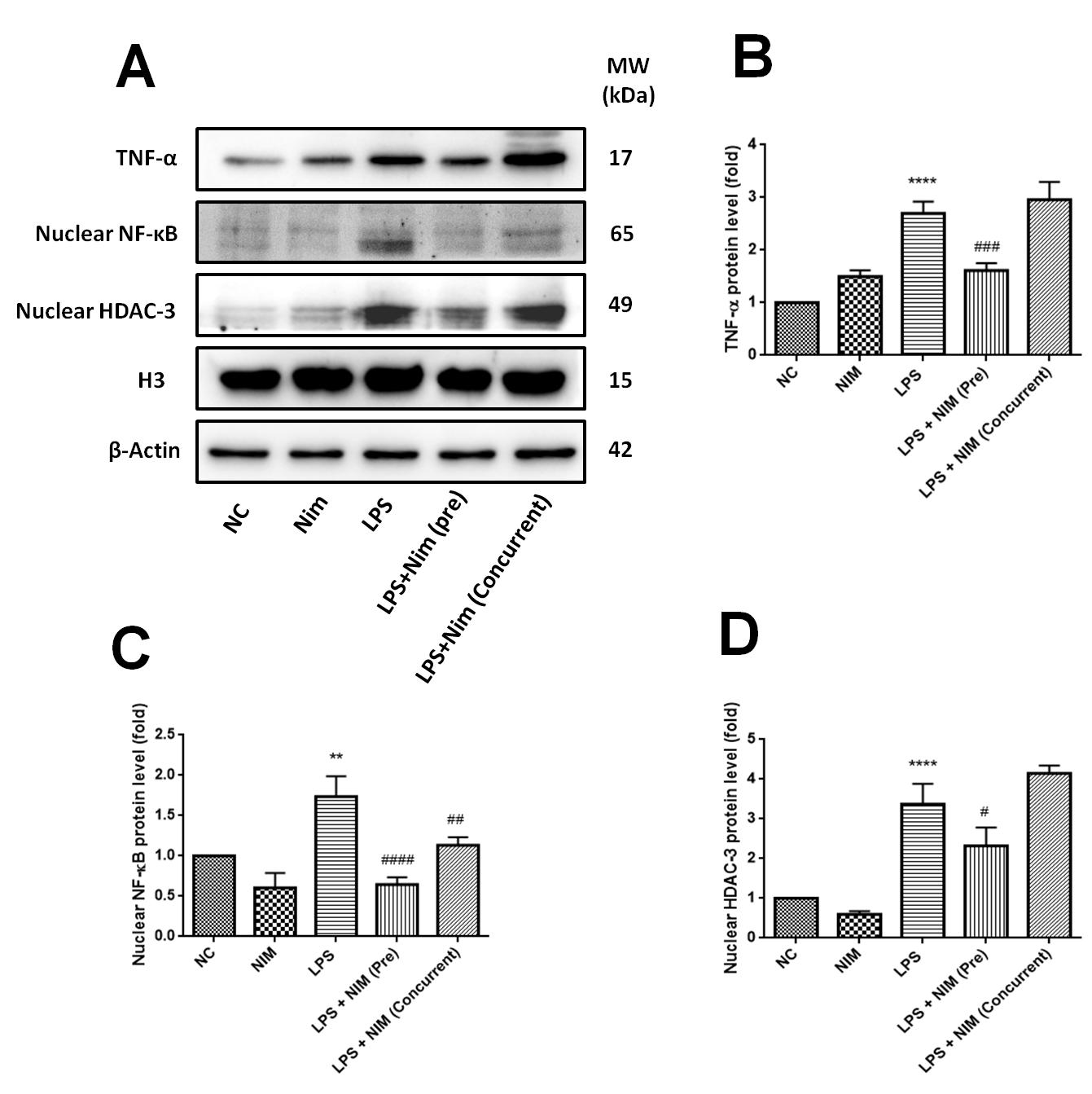


**Figure S11. Nimbolide inhibits the expression of inflammation regulated proteins in lung tissues.** Mice were pre-treated with nimbolide (3 mg/kg) for 5 days followed by LPS instillation for 12 h. In concurrent group, the animals were received the nimbolide (3 mg/kg) immediately followed by LPS instillation. (**A**) Protein was isolated and western blot analysis was performed to determine the expression of TNF-α, NF-κB, and HDAC-3. The densitometry analysis was performed by ImageJ software and protein levels of (B) TNF-α, (C) NF-κB and (D) HDAC-3 expressed as the fold change over NC. Data presented as mean ± SEM (n=8 animals per group). ***P*<0.01 and *****P*<0.0001 are significantly different from the NC group; ^#^*P*<0.05, ^##^*P*<0.01, ^###^*P*<0.001, and ^####^*P*<0.0001 are significantly different from the LPS group.


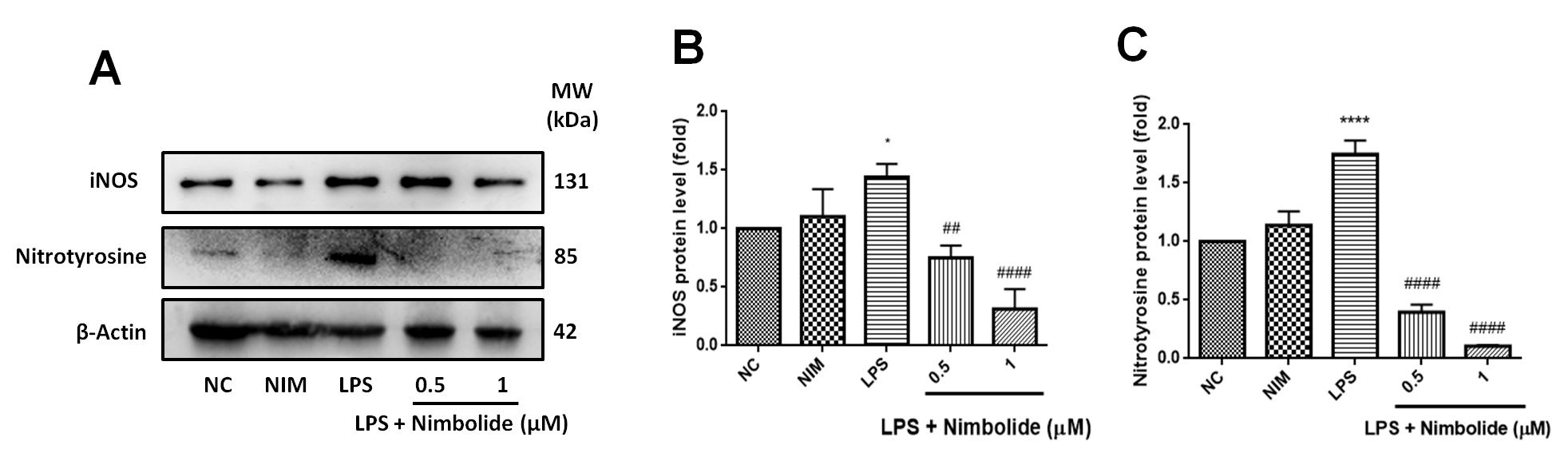


**Figure S12. Nimbolide inhibits nitrosative stress by suppressing iNOS and nitrotyrosine expression against LPS stimulation.** RAW 264.7 cells were pre-treated with nimbolide for 24 h and stimulated with LPS (1 µg/ml) for 24 h. Whereas, NIM (nimbolide alone) group (1 µg/ml) didn’t receive LPS. Protein was isolated and iNOS and nitrotyrosine expressions were determined by immunoblotting. Densitometric analysis was performed and measured the protein levels of (**B**) iNOS and (**C**) nitrotyrosine and represented as the fold change over NC. Data represented as mean ± SEM (n=3 independent experiments). **P*<0.05 and *****P*<0.0001 are significantly different from the NC group; ^##^*P*<0.01 and ^####^*P*<0.0001 are significantly different from the LPS group.

**Table S3**. GLIDE docking results and Prime MM/GBSA binding energy calculations for nimbolide and cocrystallized ligand at the active site of TNF-α protein.

| **Targeted protein** | **PDB ID** | **Ligand**  **Name** | **Docking score** | **Binding energy**  **(kcal*/*mol)** | **Interactions** | | |
| --- | --- | --- | --- | --- | --- | --- | --- |
|  |  |  |  |  | **H- bonds** | **π – π** | **Hydrophobic** |
| TNF-α | 2AZ5 | Nimbolide | -6.863 | -82.175 | Leu120, Ser60 | Tyr59 | Leu57, Tyr59, Tyr119, Leu120, Val123, Tyr151, Ile155 |
|  |  | Cocrystal | -6.513 | -64.831 | Leu120 | Tyr59 | Leu57, Ile58,Tyr59, Tyr119, Leu120, Val123, Tyr151, Ile155 |

**REFERENCES**

1. He, M. M. *et al.* Small-molecule inhibition of TNF-alpha. *Science* **310**, 1022–1025 (2005).
